# Purification of Propylene and Ethylene by a Robust Metal–Organic Framework Mediated by Host–Guest Interactions

**DOI:** 10.1002/anie.202103936

**Published:** 2021-06-07

**Authors:** Jiangnan Li, Xue Han, Xinchen Kang, Yinlin Chen, Shaojun Xu, Gemma L. Smith, Evan Tillotson, Yongqiang Cheng, Laura J. McCormick McPherson, Simon J. Teat, Svemir Rudić, Anibal J. Ramirez‐Cuesta, Sarah J. Haigh, Martin Schröder, Sihai Yang

**Affiliations:** ^1^ Department of Chemistry The University of Manchester Manchester M13 9PL UK; ^2^ Department of Materials The University of Manchester Manchester M13 9PL UK; ^3^ Neutron Scattering Division Neutron Sciences Directorate Oak Ridge National Laboratory Oak Ridge TN 37831 USA; ^4^ Advanced Light Source Lawrence Berkeley National Laboratory Berkeley CA 94720 USA; ^5^ ISIS facility, Science and Technology Facilities Council (STFC) Rutherford Appleton Laboratory Didcot OX11 0QX UK

**Keywords:** crystallography, ethylene, host–guest interactions, metal–organic framework, propylene

## Abstract

Industrial purification of propylene and ethylene requires cryogenic distillation and selective hydrogenation over palladium catalysts to remove propane, ethane and/or trace amounts of acetylene. Here, we report the excellent separation of equimolar mixtures of propylene/propane and ethylene/ethane, and of a 1/100 mixture of acetylene/ethylene by a highly robust microporous material, MFM‐520, under dynamic conditions. In situ synchrotron single crystal X‐ray diffraction, inelastic neutron scattering and analysis of adsorption thermodynamic parameters reveal that a series of synergistic host–guest interactions involving hydrogen bonding and π⋅⋅⋅π stacking interactions underpin the cooperative binding of alkenes within the pore. Notably, the optimal pore geometry of the material enables selective accommodation of acetylene. The practical potential of this porous material has been demonstrated by fabricating mixed‐matrix membranes comprising MFM‐520, Matrimid and PIM‐1, and these exhibit not only a high permeability for propylene (≈1984 Barrer), but also a separation factor of 7.8 for an equimolar mixture of propylene/propane at 298 K.

## Graphical abstract

A comprehensive understanding of the host–guest chemistry of MFM‐520 at a molecular level rationalises the observed high selectivity towards olefins, and informs future designs of improved materials for challenging olefin/paraffin separations in industry.

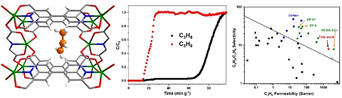

## Introduction

Over 200 million tonnes of ethylene (C_2_H_4_) and propylene (C_3_H_6_) are produced from steam cracking of naphtha each year, consuming 0.3 % of the global energy production.^[1]^ The downstream purification to produce polymer‐grade (>99.9 %) olefins is based upon cryogenic distillation. This is a highly energy‐intensive process primarily due to the requirements of cooling and compressing mixed hydrocarbon streams at an enormous scale.[^2^, ^3^] However, this is insufficient to remove trace amounts of acetylene, an impurity in olefin streams which irreversibly poisons polymerisation catalysts. Furthermore, any build‐up of acetylene can be explosive.^[2]^ Removal of acetylene by its partial hydrogenation to ethylene over supported palladium‐catalysts is a widely used solution, but suffers from poor selectivity and very high cost.^[4]^

By exploiting their active sites,^[5]^ functional groups,[^6^, ^7^] pore sizes^[8]^ and geometry,^[9]^ metal‐organic framework (MOF) materials can show preferential adsorption of alkynes over alkenes,[^6^, ^9^, ^10^, ^11^] and alkenes over alkanes.[^5^, ^8^, ^12^] MOFs incorporating open metal sites afford highly selective binding of unsaturated hydrocarbons, typically by forming a co‐ordination complex; however, such systems are often sensitive to moisture and the regeneration of sorbent is not always straightforward. MOFs that incorporate suitable narrow pores can achieve remarkable adsorption selectivities owing to molecular sieving effects. For example, UTSA‐280 excludes C_2_H_6_ molecules and exhibits a C_2_H_4_/C_2_H_6_ selectivity of >10 000, setting a new benchmark for C_2_H_4_ purification.^[8]^ Similarly, UTSA‐200a,^[13]^ ELM‐11,^[14]^ ELM‐13,^[14]^ UTSA‐300a^[15]^ and NTU‐65^[16]^ all display exclusion of C_2_H_4_ and show high selectivities of C_2_H_2_/C_2_H_4_. Recently, a synergistic sorbent separation technology for the one‐step production of polymer‐grade C_2_H_4_ from ternary (C_2_H_2_/C_2_H_6_/C_2_H_4_) and quaternary (CO_2_/C_2_H_2_/C_2_H_6_/C_2_H_4_) gas mixtures has been reported by integrating a series of MOFs with varying selectivities into a fixed‐bed.^[17]^ In contrast, reports on the separation of C_3_H_6_ and C_3_H_8_ by porous materials is limited. To date, selective adsorption of C_3_H_6_ over C_3_H_8_ has been achieved via binding of the unsaturated component, C_3_H_6_, to open metal sites as in MOF‐74(Fe),^[5]^ by molecular exclusion of C_3_H_8_ in KAUST‐7,^[18]^ Y‐abtc^[19]^ and Co‐gallate,^[20]^ by differences in adsorption kinetics in MOFs adopting narrow pores,[^21^, ^22^, ^23^, ^24^] or by equilibrium‐kinetic synergetic effects.^[25]^

Here, we report the efficient separation of equimolar mixtures of C_3_H_6_/C_3_H_8_ and C_2_H_4_/C_2_H_6_, and a 1:100 mixture of C_2_H_2_/C_2_H_4_ by a microporous MOF, MFM‐520, to produce polymer‐grade C_2_H_4_ and C_3_H_6_ at 318 K. The chosen temperature is close to that (313 K) of the mixed hydrocarbon stream for compression in cracking processes,^[26]^ thus potentially saving more energy than those working at room temperature. We have used in situ synchrotron single crystal X‐ray diffraction (SSCXRD) and inelastic neutron scattering (INS) to unravel the details of the host‐guest binding at molecular resolution to confirm that a combination of optimal pore size, geometry and pore interior chemistry (aryl pockets) underpins the observed efficient separations of mixtures of alkyne/alkene and alkene/alkane in MFM‐520. The absence of open metal sites results in facile regeneration of the sorbent under pressure‐swing conditions, and the material additionally shows high stability towards water. A ternary mixed‐matrix membrane (MMM) comprised of PIM‐1/Matrimid/MFM‐520 (*w*/*w*/*w*=10:10:1) shows a permeability for C_3_H_6_ and a separation factor for C_3_H_6_/C_3_H_8_ both of which surpasses the current upper bound for C_3_H_6_/C_3_H_8_ separation, thus demonstrating the practical potential of MFM‐520 for the purification of olefins.

## Results and Discussion

**Figure 1:**
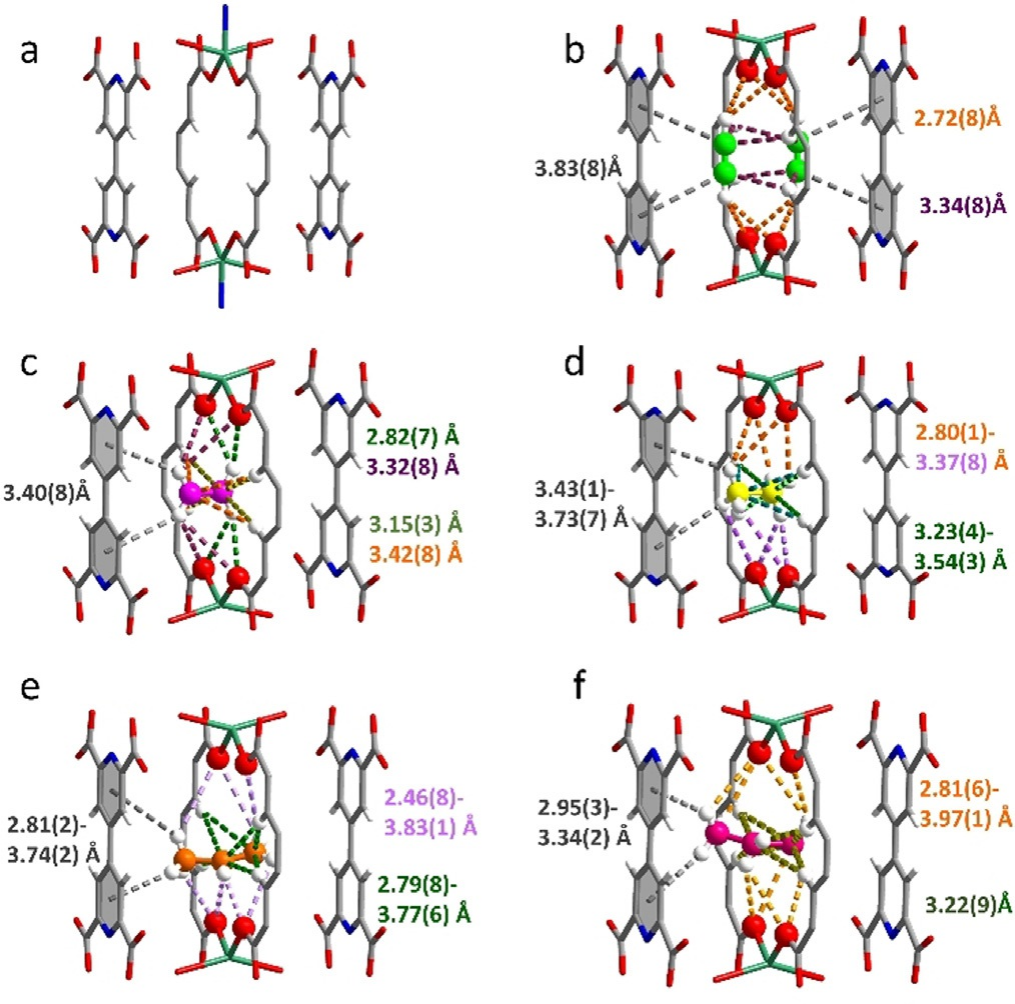
Views of the crystal structures of bare MFM‐520 and the C_2_H_2_‐, C_2_H_4_‐, C_2_H_6_‐, C_3_H_6_‐ and C_3_H_8_‐adsorbed MFM‐520 (C: grey; N: blue; O: red; H: white; Zn: dark green). Only one cavity of dimension 6.6×4.0×3.6 Å is shown. Each unit cell contains two such cavities. All structures were obtained by refinement of SSCXRD data collected at 273 K. Structure of a) bare MFM‐520; b) C_2_H_2_‐loaded MFM‐520 (C of C_2_H_2_: green); c) C_2_H_4_‐loaded MFM‐520 (C of C_2_H_4_: magenta); d) C_2_H_6_‐loaded MFM‐520 (C from C_2_H_6_, yellow); e) C_3_H_6_‐loaded MFM‐520 (C of C_3_H_6_: orange) and f) C_3_H_8_‐loaded MFM‐520 (C of C_3_H_8_: pink). The colour of each distance refers to the interaction of the same colour.

**Figure 2:**
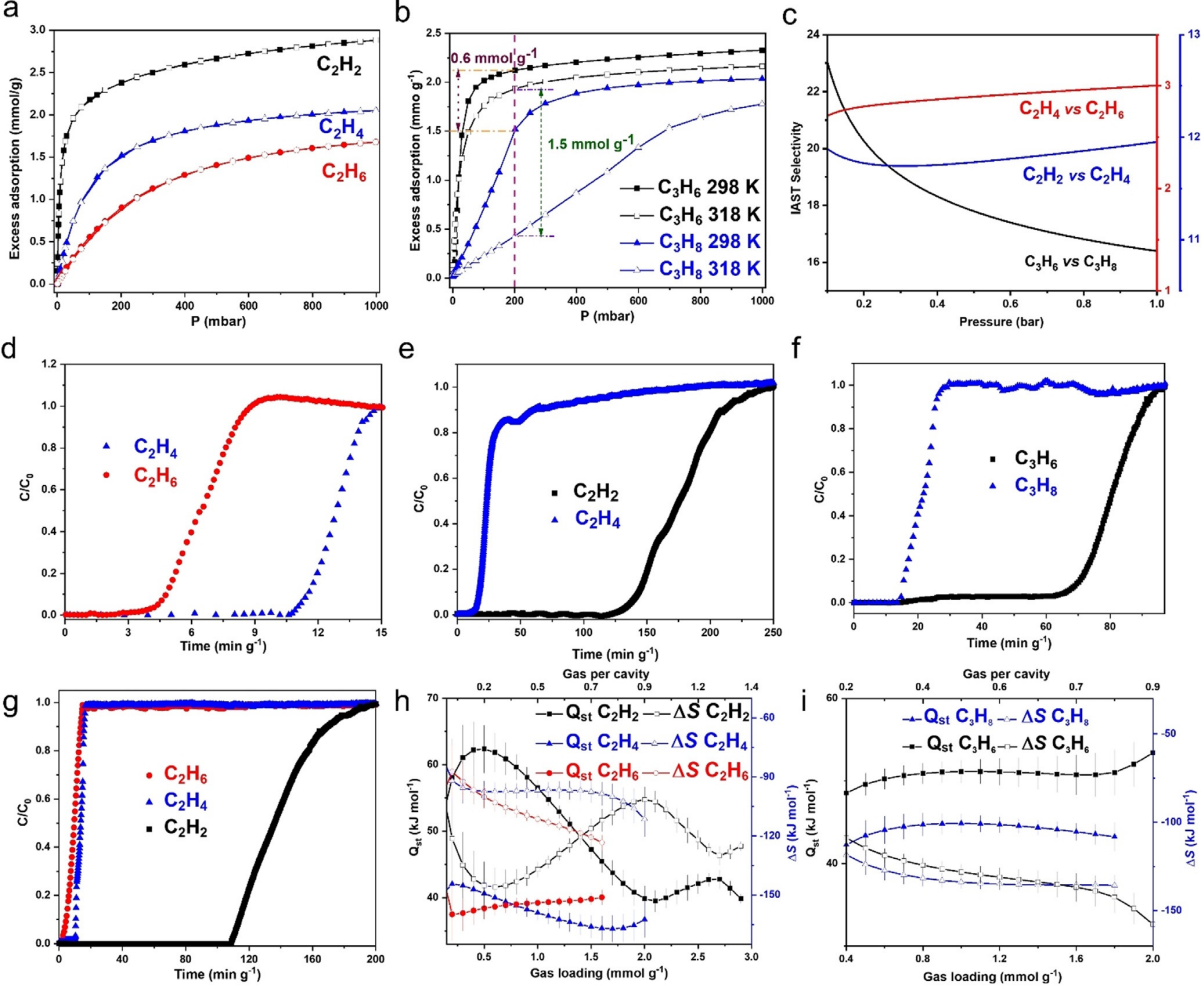
Adsorption isotherms, selectivity, thermodynamics and dynamic separation data. Views of a) adsorption isotherms for C_2_H_2_, C_2_H_4_ and C_2_H_6_ in MFM‐520 at 318 K (adsorption: solid; desorption: open symbols); b) adsorption isotherms of C_3_H_6_ and C_3_H_8_ in MFM‐520 at 298 and 318 K; desorption isotherms are omitted for clarity; c) IAST selectivities of the equimolar mixtures of C_2_H_4_/C_2_H_6_ and C_3_H_6_/C_3_H_8_, and of a 1:100 mixture of C_2_H_2_/C_2_H_4_ at 0.1–1.0 bar in MFM‐520 at 318 K; breakthrough plots for d) an equimolar mixtures of C_2_H_4_/C_2_H_6_, e) a 1:100 mixture of C_2_H_2_/C_2_H_4_, f) an equimolar mixture of C_3_H_6_/C_3_H_8_, and g) a ternary mixture of C_2_H_2_/C_2_H_4_/C_2_H_6_ (1:100:100) at 318 K with a flow rate of 4–6 mL min^−1^; variation of *Q_st_* and *ΔS* for uptakes of h) C_2_ hydrocarbons and i) C_3_ hydrocarbons in MFM‐520 (black square: C_2_H_2_, C_3_H_6_; blue triangle: C_2_H_4_, C_3_H_8_; red circle: C_2_H_6_; solid: *Q_st_* and open: Δ*S*). Full isotherm data are shown in the Supporting Information.

MFM‐520 was chosen for the study of hydrocarbon separation because of its bowtie‐shaped cavity with suitable dimensions of 6.6×4.0×3.6 Å (Figure 1 a) and its high structural stability.[^27^, ^28^] Desolvated MFM‐520 displays a three‐dimensional 4^4^6^6^‐connected framework structure with a *sqp* ^[29]^ topology and a BET (Brunauer, Emmett and Teller) surface area of 313 m^2^ g^−1^. Gravimetric adsorption isotherms of light hydrocarbons were measured at 273–318 K and up to 1 bar (Figures 2 a,b and Figures S1–5). MFM‐520 displays fully reversible uptakes of 3.09, 2.36, 1.93, 2.33 and 2.03 mmol g^−1^ for C_2_H_2_, C_2_H_4_, C_2_H_6_, C_3_H_6_ and C_3_H_8_, respectively, at 298 K and 1 bar. Interestingly, while the adsorption capacity of C_2_H_6_ and C_3_H_8_ in MFM‐520 decreases rapidly with the increasing temperatures, consistent with majority of reported adsorption isotherms for MOFs, the variation of temperature has a much smaller effect on the uptake of C_2_H_2_, C_2_H_4_ and C_3_H_6_, particularly in the low pressure region where only small changes are observed for C_3_H_6_ adsorption. For example, the uptakes of C_3_H_6_ at 200 mbar are 2.12 and 1.93 mmol g^−1^ at 298 and 318 K, respectively, whereas for C_3_H_8_ these are 1.51 and 0.43 mmol g^−1^ under the same conditions. Thus, the difference (0.61 and 1.50 mmol g^−1^ at 298 and 318 K, respectively) in adsorption capacity of C_3_H_8_ and C_3_H_6_ of MFM‐520 is significantly amplified at 318 K (Figure 2 b). Analysis of the single‐component isotherms at 318 K using ideal adsorbed solution theory (IAST)^[30]^ yields selectivities of 3.0, 23‐17 and ≈12 for the equimolar mixtures of C_2_H_4_/C_2_H_6_ and C_3_H_6_/C_3_H_8_, and for a 1:100 mixture of C_2_H_2_/C_2_H_4_, respectively (Figure 2 c).

Dynamic breakthrough experiments were conducted by flowing equimolar mixtures of C_3_H_6_/C_3_H_8_ and C_2_H_4_/C_2_H_6_, and a 1:100 mixture of C_2_H_2_/C_2_H_4_ through a fixed‐bed packed with MFM‐520 at 318 K and 1 bar (Figures 2 d–f). Excellent separations were achieved in all cases. For example, MFM‐520 shows a rapid breakthrough of C_3_H_8_ with selective retention of C_3_H_6_ (retention time of 15 and 68 min g^−1^, respectively). The 1:100 mixture of C_2_H_2_/C_2_H_4_ displays an almost immediate breakthrough of C_2_H_4_ with highly selective removal of C_2_H_2_ (retention time of 11 and 125 min g^−1^, respectively). The high retention of C_2_H_2_ enables production of high‐purity C_2_H_4_ (>99.9 %) at the outlet. Importantly, an excellent separation has also been achieved for the separation of a ternary mixture of C_2_H_6_/C_2_H_4_/C_2_H_2_ (100:100:1) which shows retention times of 1.5, 9.0, and 110 min g^−1^, respectively; (Figure 2 g). The fixed‐bed of MFM‐520 can be readily regenerated by flowing He or applying dynamic vacuum for 1 h at 318 K. The separation performance of MFM‐520 compares favourably with leading MOFs in the literature (Table S1).

In situ SSCXRD of MFM‐520 as a function of gas loading at 273 K reveals the preferred binding domains for C_2_H_2_, C_2_H_4_, C_2_H_6_, C_3_H_6_ and C_3_H_8_ in the cavity (Figure 1 b–f). The low temperature was chosen to minimize the thermal disorder of adsorbed guest molecules, and the crystallographic uptakes are generally consistent with those recorded in isotherms. Each cavity (6.6×4.0×3.6 Å) can accommodate two molecules of C_2_H_2_, but only one molecule for all the other gases owing to the smaller molecular size of C_2_H_2_, consistent with the higher adsorption uptake observed for C_2_H_2_. The C−C and C−H bond distances of adsorbed C_2_H_2_ are 1.11(3) and 0.93(7) Å, respectively, with ∡ H−C−C=179.9(3)°, confirming the absence of significant molecular distortion on binding. Each adsorbed C_2_H_2_ molecule binds to the oxygen centre of the framework carboxylate group via a four‐fold hydrogen bonds [CH⋅⋅⋅O=2.72(8) Å, 4×], which are supplemented by parallel π⋅⋅⋅π stacking interactions between the π‐electrons of C_2_H_2_ molecules and pyridyl rings in a {pyridine⋅⋅⋅C_2_H_2_⋅⋅⋅C_2_H_2_⋅⋅⋅pyridine} sequence [distances of 3.83(8), 2.96(9) and 3.83(8) Å, respectively]. Each C_2_H_2_ molecule is further surrounded by four hydrogen atoms of the pyridine rings, forming weak supramolecular interactions [HC(C_2_H_2_)⋅⋅⋅HC(pyridine)=3.34(8) Å]. Thus, each C_2_H_2_ molecule is stabilised by a 10‐fold host‐guest interaction in a highly cooperative manner within the aryl and oxygen‐rich cavity of MFM‐520. Weak intermolecular interactions are also observed between the two C_2_H_2_ molecules within the same cavity [HC(C_2_H_2_)⋅⋅⋅HC(C_2_H_2_)=3.11(3) Å]. The accuracy of interaction regions were further confirmed by Hirshfeld surface analysis (Figure S21).

Adsorbed C_2_H_4_, C_2_H_6_, C_3_H_6_ and C_3_H_8_ molecules are all rotated by 90° compared to the position of the C_2_H_2_ molecule within the pore, and reside at the centre of the cavity surrounded by four hydrogen atoms from the aromatic rings, four carboxylate oxygen centres and four pyridyl rings (Figure 1 c–f). C_2_H_4_ forms two types of four‐fold hydrogen bonds with the carboxylate oxygen centre [CH⋅⋅⋅O=2.82(7), 3.32(8) Å] and with the aromatic ‐CH groups [C(C_2_H_4_)⋅⋅⋅HC=3.15(3)–3.42(8) Å]. In addition, the ‐CH group of C_2_H_4_ interacts with the pyridyl ring [CH(C_2_H_4_)⋅⋅⋅ring centroid=3.40(8) Å]. Adsorbed C_2_H_6_ molecules show longer host‐guest binding distances overall [CH⋅⋅⋅O=2.80(1)–3.37(8) Å; C(C_2_H_6_)⋅⋅⋅HC=3.23(4)–3.54(3) Å; CH(C_2_H_6_)⋅⋅⋅ring centroid=3.43(1)–3.73(7) Å]. Interestingly, adsorbed C_3_H_6_ molecules show notably shorter host‐guest interactions compared with C_3_H_8_, particularly for the hydrogen bonds to the carboxylate oxygen centres [CH⋅⋅⋅O=2.46(8)–3.83(1); 2.81(6)–3.97(1) Å, respectively] and for the supramolecular interactions between the C=C bond and the aromatic hydrogen atoms [C(C_3_H_6_)⋅⋅⋅HC=2.79(8) Å; C(C_3_H_8_)⋅⋅⋅HC=3.22(9) Å, respectively]. The structures reveal unambiguously the molecular details of the host‐guest interactions, entirely consistent with the observed selective retention of C_2_H_2_, C_2_H_4_ and C_3_H_6_ in the breakthrough separations of mixtures of C_2_H_2_/C_2_H_4_, C_2_H_4_/C_2_H_6_ and C_3_H_6_/C_3_H_8_, respectively.

The isosteric heat of adsorption (*Q_st_*) and entropy of adsorption (Δ*S*) for all hydrocarbons were calculated from the adsorption isotherms recorded at different temperatures (Figure 2 h, i, S6, S15, Table S3–S7). C_2_H_2_ displayed a value for *Q_st_* of 60 kJ mol^−1^ at low surface coverage, which steadily decreases to ≈40 kJ mol^−1^ with increasing loading. Interestingly, Δ*S* for the uptake of C_2_H_2_ shows an unusual increase on loading between 0.2 and 0.9 molecule per cavity, indicating the presence of an increased disorder of the host‐guest system that plays a positive role in the adsorption. This is likely caused by the random distribution of each C_2_H_2_ molecule between two available sites within the pore (Figure 1 b). This is consistent with the observed small increase of *Q_st_* above the loading of ≈1.0 molecule per cavity, indicating the presence of additional, weak intermolecular interactions between adsorbed C_2_H_2_ molecules within each cavity. The *Q_st_* of adsorption for C_2_H_4_ and C_2_H_6_ are both around 40 kJ mol^−1^, notably lower than that of C_2_H_2_ and these show little change with loading. Values of Δ*S* show a steady decrease on uptake of C_2_H_4_ and C_2_H_6_. C_3_H_6_ and C_3_H_8_ display similar trends in *Q_st_* and Δ*S* on gas loading and the former shows a higher value of *Q_st_* due to interactions of the unsaturated C=C bond and the host. Analysis of these thermodynamic parameters is entirely consistent with the observed selective adsorption of C_2_H_2_ and C_3_H_6_.

**Figure 3:**
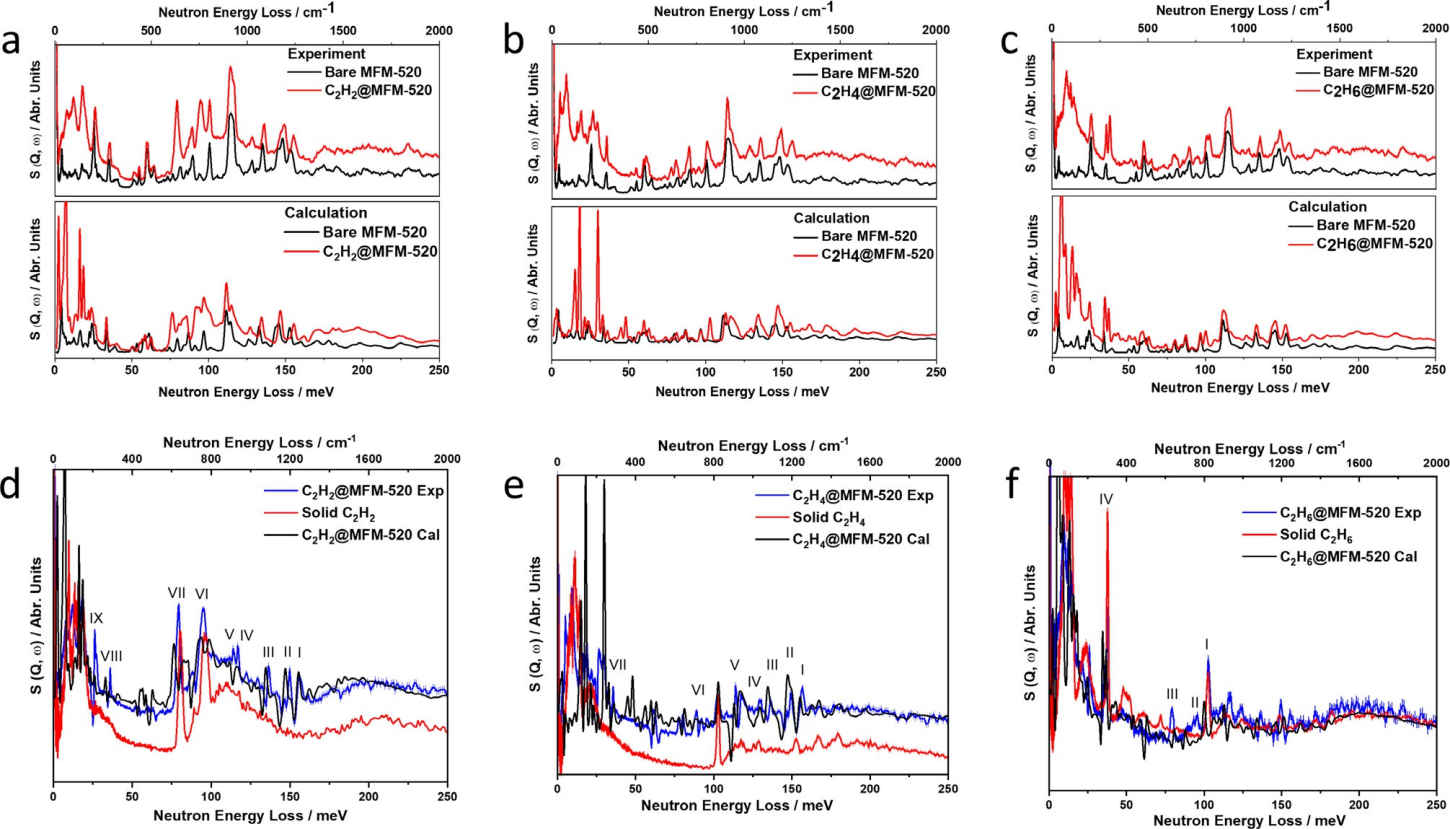
INS spectra of MFM‐520 as a function of hydrocarbon loadings. a–c) Comparison of the experimental (top) and DFT‐calculated (bottom) INS spectra for bare MFM‐520 and MFM‐520 loaded with (a) C_2_H_2_ (b) C_2_H_4_ and (c) C_2_H_6_; d–f) comparison of difference plots for experimental and DFT‐calculated INS spectra of bare MFM‐520 and MFM‐520 loaded with (d) C_2_H_2_, (e) C_2_H_4_ and (f) and C_2_H_6_, and the experimental INS spectra of condensed (d) C_2_H_2_, (e) C_2_H_4_ and (f) C_2_H_6_ in the solid state.

Combined INS and DFT investigations enabled the direct visualisation of binding dynamics of adsorbed C_2_H_2_, C_2_H_4_ and C_2_H_6_ molecules within MFM‐520. The INS spectra were collected at 7 K (Figure 3) to minimise the thermal motion of hydrocarbon molecules and the host. DFT calculations used the structural models obtained from SSCXRD experiments to enable assignment of vibrational features, and the averaging of positionally disordered molecules in the calculations accounts for the small discrepancies observed between experiment and calculation. In the difference spectra, nine major changes appear upon loading C_2_H_2_ into desolvated MFM‐520. Peaks I to VII occur at high energy (156 to 75 meV) and peaks VIII and IX at low energy (35 and 26 meV, respectively). Peaks I (156 meV), III (137 meV) and V (114 meV) are assigned to the symmetric, asymmetric and out‐of‐plane bending modes of the framework ‐CH groups, and the notable changes of these peaks suggest strong H_2_C_2_⋅⋅⋅HC‐(pyridine) interactions. Peaks II (150 meV), IV (117 meV), VIII (35 meV) and IX (26 meV) are associated with various ring deformation and lattice modes, which are consistent with the formation of π⋅⋅⋅π stacking interactions. Peaks VI (92 meV) and VII (77 meV) are assigned to *syn*‐ and *anti*‐ C−H bending modes of adsorbed C_2_H_2_ molecules; compared with those of the solid C_2_H_2_ (97, 81 meV, respectively), the red‐shifts of these peaks by 4–5 meV (or 32–40 cm^−1^) indicate reduced strength of the internal modes of C_2_H_2_ upon formation of hydrogen bonding to the carboxylate groups of MFM‐520. To the best of our knowledge, such red‐shifts have not been previously observed for adsorbed C_2_H_2_ molecules in porous solids and demonstrate their tight confinement in MFM‐520.

Seven features were observed on the difference spectrum obtained by subtracting the spectrum of bare MFM‐520 from that of C_2_H_4_‐loaded MFM‐520. Peaks I (156 meV), III (137 meV) and V (114 meV) can be assigned to the symmetric, asymmetric and out‐of‐plane bending modes of the framework ‐CH groups, consistent with H_4_C_2_⋅⋅⋅HC‐(pyridine) interactions. Peaks II (150 meV), VI (89 meV) and VII (35 meV) are associated with various rings deformation and lattice modes, which are consistent with the formation (C_2_H_4_)‐CH_2_⋅⋅⋅pyridyl ring interactions. Changes of peak IV (129 meV) assigned to the out‐of‐plane bending mode of ‐CH_2_ group in C_2_H_4_ are consistent with the formation of hydrogen bonds between C_2_H_4_ and carboxylate oxygen centres. For C_2_H_6_ loading into MFM‐520, weaker host‐guest interactions are expected and the changes of peak intensity and energy are indeed less pronounced. Indeed, peaks I (102 meV) and IV (38 meV), which are assigned to the bending and torsion modes of ‐CH_3_ groups in adsorbed C_2_H_6_ molecules, respectively, show negligible shifts. Small changes at peaks II (93 meV) and III (80 meV), associated with the out‐of‐plane and in‐plane bending modes of framework ‐CH group, respectively, indicate very weak C_2_H_6_‐framework interactions. Thus, the combination of crystallography and INS studies reveal the host‐guest binding dynamics of hydrocarbon‐loaded MFM‐520, and directly support the observed selectivity in gas separation experiments.

**Figure 4:**
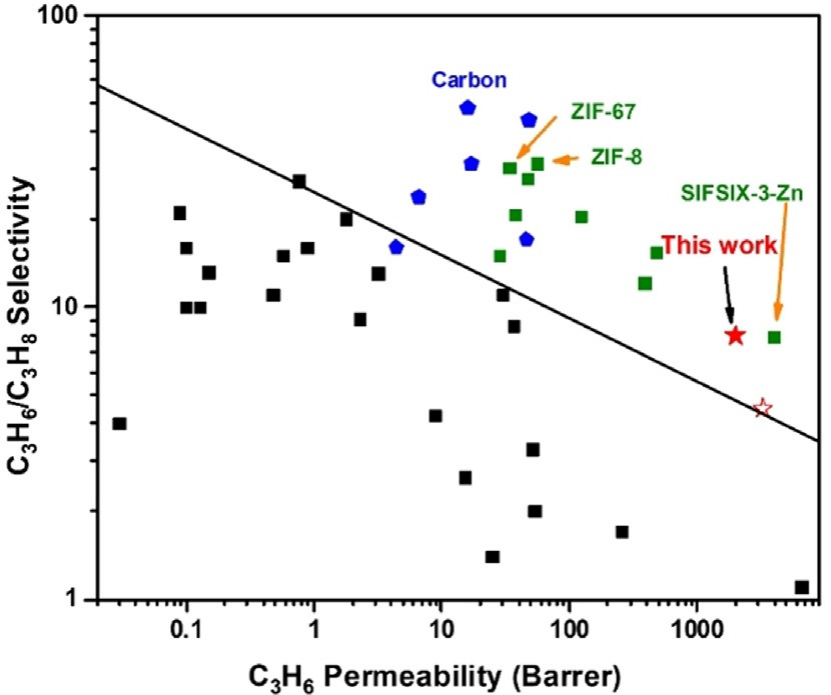
Performance of C_3_H_6_/C_3_H_8_ separation of selected polymers (black square), carbons (blue pentagon), MOF/ZIF‐based MMMs (olive square), MFM‐520 MMM (solid red star) and PIM‐1/Matrimid (open red star). Some reported data are based on measurements of permeation of single gas. Details are given in the Supporting Information Table S10. Solid line represents the experimentally observed upper bound for C_3_H_6_/C_3_H_8_ separation within the polymer membranes.

Membrane‐based separation techniques are widely considered to be energy‐efficient alternatives to traditional distillation processes.[^31^, ^32^, ^33^] MMMs can effectively improve the trade‐off between selectivity and permeability in pure polymer‐based membranes by incorporating porous fillers.^[33]^ Although polymer‐based thin‐films and MMMs have been studied intensively for the separation of various gas mixtures, such as H_2_/CO_2_,[^34^, ^35^, ^36^] CO_2_/N_2_,^[37]^ CO_2_/CH_4_[^38^, ^39^, ^40^] and O_2_/N_2_,^[41]^ polymer‐based membranes have shown limited separation factors or permeability[^42^, ^43^, ^44^] for the separation of C_3_H_6_/C_3_H_8_, and such studies based upon MOF‐incorporated MMMs have only been reported in limited cases, such as ZIF‐8, ZIF‐67 and SIFSIX[^32^, ^45^, ^46^, ^47^, ^48^] (Figure 4, Table S10). We sought to fabricate MMMs based upon MFM‐520 and study their performance in the separation of C_3_H_6_/C_3_H_8_. PIM‐1 (polymers of intrinsic microporosity) are a mature technology with a superior gas permeability[^49^, ^50^] and commercial Matrimid possesses prominent selectivity for gas‐pairs, high thermal stability and good processability,[^51^, ^52^] making them good candidates as the support to MMMs. A ternary MMM, PIM‐1/Matrimid/MFM‐520 (*w*/*w*/*w*=10:10:1) and a binary membrane (PIM‐1/Matrimid, *w*/*w*=1:1) were fabricated and exhibited good flexibility. Retention of the structure of MFM‐520 in the MMM was confirmed by PXRD (Figure S16), and scanning electron microscopy (SEM) images showed a homogenous texture for the MMM, implying a homogenous distribution of MOF throughout the membrane (Figure S17). The permeation of C_3_H_6_ and C_3_H_8_ was measured at 1.5 bar and 298 K, and the ternary MMM displays a high separation factor of 7.8 and a permeability for C_3_H_6_ of ≈1984 Barrer (Figure 4). This performance is better than that of the binary polymer membrane, which shows a separation factor of 4.4 and a permeability for C_3_H_6_ of ≈3242 Barrer). This confirms that MFM‐520 plays a key role in the dynamic separation of the as‐formed MMM. Thus, by improving the permeability of Matrimid and the selectivity of PIM‐1, the MMM based upon PIM‐1/Matrimid/MFM‐520 exhibits superior performance that surpasses the current upper bound for C_3_H_6_/C_3_H_8_ separation and compares favourably with other MOF‐containing MMMs.

## Conclusion

Powerful drivers exist for the development of efficient separation techniques to purify lower olefins. Regenerable porous solid sorbents possessing high selectivity and stability are highly desirable. Fundamental understanding of the host‐guest binding at a molecular level provides important insights to guide the design of new materials with improved properties. In this study, we have investigated comprehensively the preferred adsorption domain and host‐guest binding dynamics of MFM‐520 on loadings of various C_2_ and C_3_ hydrocarbons at crystallographic resolution by in situ SSCXRD and INS, coupled with DFT modelling and analysis of adsorption thermodynamic parameters. The highly confined pore of MFM‐520 differentiate between alkenes from alkanes by fine‐tuning of the host‐guest interactions in the presence of C=C bonds in alkenes as a function of temperature, and an optimal separation has been achieved at 318 K, a temperature that is relevant to the compression of mixed hydrocarbons in cracking processes. The unique pore geometry of MFM‐520 enables the selective uptake of acetylene over ethylene, thus resulting in the effective removal of trace acetylene and the production of polymer‐grade ethylene. Along with its ultra‐high stability against water and air, the practical potential of MFM‐520 has also been demonstrated by both column breakthrough and MMM separations.^[53]^

## Conflict of interest

The authors declare no conflict of interest.

## Supporting information

As a service to our authors and readers, this journal provides supporting information supplied by the authors. Such materials are peer reviewed and may be re‐organized for online delivery, but are not copy‐edited or typeset. Technical support issues arising from supporting information (other than missing files) should be addressed to the authors.

Supplementary
