# Supplementary material for: Purification of Propylene and Ethylene by a Robust Metal–Organic Framework Mediated by Host–Guest Interactions
Source: Angew Chem Int Ed Engl. 2021 Jun 7;60(28):15541–7. doi: 10.1002/anie.202103936 (PMC8362173; doi:10.1002/anie.202103936)
Supplement: Supplementary file 1 — Supplementary [file ANIE-60-15541-s001.pdf]

Angewandte Chemie
Eine Zeitschrift der Gesellschaft Deutscher Chemiker

Supporting Information

Purification of Propylene and Ethylene by a Robust Metal–Organic Framework Mediated by Host–Guest Interactions

Jiangnan Li, Xue Han, Xinchen Kang, Yinlin Chen, Shaojun Xu, Gemma L. Smith, Evan Tillotson, Yongqiang Cheng, Laura J. McCormick McPherson, Simon J. Teat, Svemir Rudić, Anibal J. Ramirez-Cuesta, Sarah J. Haigh, Martin Schröder, and Sihai Yang**

anie_202103936_sm_miscellaneous_information.pdf

Supplementary Information

Table of Contents

Materials and Methods S2
Additional Gas Adsorption Isotherms for MFM-520 .... S5
Summary of Materials Reported for Olefin Purification S10
Summary of Crystallographic Data for Hydrocarbon-loaded MFM-520 .... S11
Isosteric Heats of Adsorption S12
PXRD Analysis of PIM-1/Matrimid/MFM-520 (w/w/w =10: 10: 1) MMM S27
SEM Analysis of PIM-1/Matrimid/MFM-520 (*w*/*w*/*w* = 10:10:1) MMM S28
Optical Photographs of Fabricated Membranes S29
The Reproducibility of the Separation Performance of the PIM-1/Matrimid/MFM-520 MMM S30
The Reproducibility of the Separation Performance of the PIM-1/Matrimid Membrane S31
Summary of Selected C_3H_6/C_3H_8 Separations using Membranes S32
IAST analysis at 298 K S33
View of Packing of Adsorbed C_2H_2 Molecules in MFM-520 .... S34
SI References S36

Materials and Methods

Preparation of MFM-520

All reagents were used as received from commercial suppliers without purification. The syntheses of H_4L ligand, $\{[Zn_2(L)\cdot 4H_2O]\}_\infty$(MFM-520-solvate) and $[Zn_2(L)]_\infty$(MFM-520) were carried out using our previously reported method[1].

Gas Adsorption Isotherms

Gravimetric sorption isotherms were collected on the IGA-003 system (Hiden Isochema, Warrington, UK) under ultra-high vacuum produced by a turbo pumping system and the

temperatures were controlled by system programmed water bath. Before measurement of the isotherm, the as-synthesized MFM-520 was activated at 120 °C under dynamic high vacuum (10^{-10} bar measured at the pump) for 24 h to give the fully desolvated MFM-520. C_2H_4, C_2H_6, C_3H_6, and C_3H_8 were ultra-pure research-grade (99.999%) purchased from BOC. C_2H_2 was purified through an activated carbon filter before introduction to the IGA system.

Gas Separation Breakthrough Experiments

Breakthrough experiments were performed on a Hiden Isochema IGA-003 with ABR attachments in combination with a Hiden Analytical mass spectrometer and FTIR spectrometer to detect the gases as they are released from the sample bed. The temperature was controlled by a temperature-programmed system. Breakthrough experiments were carried out in a 7 mm diameter fixed-bed of 120 mm length packed with ~1.0 g of MFM-520 powder. The sample was heated at 120 ºC under a flow of He for 16h for activation. The fixed-bed was then cooled to 318 K using a temperature programmed water bath and the breakthrough experiment was performed with a stream of hydrocarbon gases at atmospheric pressure and 318 K. The flow rate of the entering gas mixture was maintained at 4-6 mL min^{-1}, and the gas concentration, C, of hydrocarbons at the outlet was determined by mass spectrometry and compared with the corresponding inlet concentration C_0, where $C/C_0 = 1$ indicates complete breakthrough.

Inelastic Neutron Scattering

Inelastic neutron scattering (INS) experiments were undertaken using the TOSCA spectrometer at the ISIS facility. TOSCA is an indirect geometry crystal analyser instrument that provides a wide dynamic range (-26-4000 cm^{-1}) with resolution optimised in the 50-2000 cm^{-1} range. In this region TOSCA has a resolution of 1.25% of the energy transfer. The instrument is comprised of 130 ^3He detectors in the forward and backscattering geometry located 17 m downstream of a 300 K Gd poisoned water moderator. A temperature of 7 ± 0.2 K was maintained during data collection by two He closed cycle refrigerators with 30 mbar He as an exchange gas.

MFM-520 was loaded into an 11 mm diameter vanadium sample can and degassed at 10^{-7} mbar and 120 °C for 1 day. The loading of gases was performed at room temperature and the sample can loaded into a closed cycle refrigerator (CCR) He cryostat and cooled to (7 ± 0.1 K) for data collection. C_2H_2, C_2H_4, and C_2H_6 were introduced by warming the sample to 290 K, and the gas dosed volumetrically from a calibrated volume. The gas-loaded sample was then cooled to 7 K over a period of 2 h to ensure good mobility of adsorbed gases within the crystalline structure of MFM-520. The sample was kept at 7 K for an additional 30 mins before data collection to ensure the thermal equilibrium.

DFT Modelling and Simulation

Modelling by Density Functional Theory (DFT) of the bare and $C_2H_2/C_2H_4/C_2H_6$-loaded MFM-520 was performed using the Vienna Ab initio Simulation Package (VASP)[2]. The calculation used the Projector Augmented Wave (PAW) method[3,4] to describe the effects of core electrons, and Perdew-Burke-Ernzerhof (PBE)[5] implementation of the Generalized Gradient Approximation (GGA) for the exchange-correlation functional. Energy cutoff was 800 eV for the plane-wave basis of the valence electrons. The lattice parameters and atomic coordinates determined by synchrotron X-ray single crystal diffraction in this work were used as the initial structure. The electronic structure was calculated on a 4×4×2 Monkhorst-Pack mesh for the unit cell, and at the Γ point only for the 2×2×1 supercell. The total energy tolerance for electronic energy minimization was 10^{-8} eV, and for structure optimization it was 10^{-7} eV. The maximum interatomic force after relaxation was below 0.001 eV/Å. The optB86b-vdW functional[6] for dispersion corrections was applied. The vibrational eigen-frequencies and modes were then calculated by solving the force constants and dynamical matrix using Phonopy[7]. The OClimax software[8] was used to convert the DFT-calculated phonon results to the simulated INS spectra.

Fabrication of MMM Membrane

A solution-casting method was employed for the fabrication of the mixed matrix membrane (MMM) of PIM-1/Matrimid and PIM-1/Matrimid/MFM-520. A powder of Matrimid (600 mg) was dispersed in

$CHCl_3$ (60 mL) with sonication and stirring for 12 h, and this was followed by addition of PIM-1 (600 mg) and stirring for another 12 h. When a homogenous solution was obtained, MFM-520 (60 mg) as ground powder was added and stirred for 12 h. The mixture was evaporated to 15-30 mL and cast evenly onto three PTFE substrates and the solvent evaporated at room temperature. The freestanding MMM was dried in a vacuum oven at 120 °C for 12 h to remove trace solvents from membrane prior to the separation measurements. The PIM-1/Matrimid membrane was fabricated using the same method without adding any MOF. The Matrimid was used as received from commercial suppliers without purification and the PIM-1 was synthesised following the reported method[9]. Synthesized PIM-1: GPC (in chloroform): Mw = 38,026 g mol^{-1} , and Mw/Mn = 2.309. ^1H NMR (500 MHz, $CDCl_3$, δ, ppm): 6.81 (2H, s), 6.43 (2H, s), 2.33−2.17 (4H, dd), 1.37−1.31 (broad, 12H). Anal. Calc. (Found) for $[C_{29}H_{20}N_2O_4]_n$ (wt %): C, 75.64 (74.12); H, 4.37 (4.24); N, 6.08 (6.13).

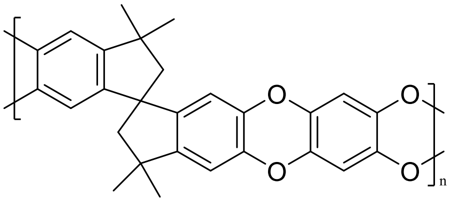

PIM-1

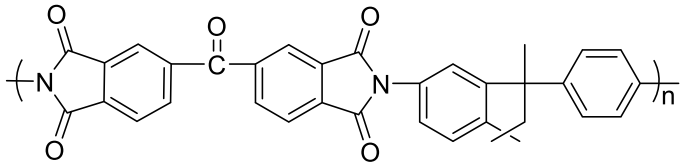

Matrimid

Gas Separation by Mixed Matrix Membrane (MMM)

Gas permeability was evaluated using the constant-pressure method with a Bruker Matrix MG5 infrared spectrometer as gas detector. The membranes of PIM-1/Matrimid and PIM-1/Matrimid/MFM-520 were activated at 120℃ under vacuum for 24 h. The permeabilities of an equimolar mixture of C_3H_6/C_3H_8 were measured at 1.5 bar and 298 K. All measurements were repeated three times to allow the standard errors to be estimated, and the membrane was activated by flushing with dry He between measurements to ensure the integrity of the membrane. A circular membrane of diameter 5.0 cm was placed in the cell. The thickness of the membrane was measured using a micrometre and multiple measurements from different sites on the membrane taken and averaged to determine the thickness. The average thickness of the PIM-1/Matrimid and PIM-1/Matrimid/MFM-520 membranes were determined to be 44 and 48 µm, respectively. The permeability was calculated by (1):

$$P = \frac{j \cdot l}{\Delta p} \qquad (1)$$

where P is the permeability coefficient in Barrer, j is the flux of the gas in 10^{-10} cm^3 (STP)/(cm^2·s), l is the thickness of the membrane in cm, and Δp is the pressure difference between the feed side and the permeate side in $cmHg^{-1}$.The selectivity for C_3H_6/C_3H_8 was calculated by equation (2)[10]:

$$\alpha C_3H_6/C_3H_8 = P(C_3H_6)/P(C_3H_8) \qquad (2)$$

***In Situ* Synchrotron Single Crystal X-Ray Diffraction**

In situ synchrotron X-ray single-crystal diffraction data were collected at beamline 11.3.1 and 12.2.1 of the Advanced Light Source in Berkeley using monochromated radiation [λ = 0.7749 and 0.7288 Å]. These *in situ* diffraction measurements were carried out in a 50 micro quartz tube coupled to

gas handling and vacuum equipment. In a typical experiment, a single crystal of synthesized MFM-520 was selected and glued onto a MiTeGen loop with Loctite Double Bubble epoxy, and the quartz tube loaded. The sample system was connected to a high vacuum (10^{-4} mbar) and heated to 420 K for ~3 h to generate desolvated MFM-520. After cooling the temperature to 273 K, the X-ray data for desolvated MFM-520 were collected as a background. No residual electron density was found in the pores of desolvated MFM-520. Upon loading of hydrocarbon gases into MFM-520 at 1 bar and 273 K for 1 h, the data were re-collected. Significant residual electron densities appeared in the pore and were sequentially assigned as adsorbed hydrocarbon molecules. Hydrogen atoms were placed and refined using a riding model, and the crystallographic data are summarized in Table S2.

Calculation of Isosteric Heats of Adsorption

To estimate the differential enthalpies (ΔH_n) and (ΔS_n) for C_2H_2, C_2H_4, C_2H_6, C_3H_6 and C_3H_8 adsorption, the isotherms at different temperatures were fitted to the van't Hoff isochore:

$$\ln(P) = \frac{\Delta Hn}{RT} - \frac{\Delta Sn}{R}$$

where *P* is pressure, *T* is the temperature, *R* is the real gas constant. Selected linear fitting plots at 1.2, 1.3, 1.4, 1.5 mmol/g are shown in Figs S7, S9, S11, S13 and S15. All linear fittings show R^2 above 0.9, indicating consistency in the isotherm data. A plot of ln(p) versus 1/T at constant amount adsorbed allows the differential enthalpy and entropy of adsorption and also the isosteric enthalpy of adsorption (Q_{st}, n) to be determined.

IAST Analysis of the Selectivity Data of $C_2H_2/C_2H_4/C_2H_6$ and C_3H_6/C_3H_8 in MFM-520

Ideal adsorbed solution theory (IAST) was used to determine the selectivity factor, *S*, for binary mixtures using pure component isotherm data. The selectivity factor, *S*, is defined according to the following Equation where x_i is the amount of each component adsorbed as determined from IAST and y_i is the mole fraction of each component in the gas phase at equilibrium. The IAST adsorption selectivity was calculated for mixtures of C_2H_2/C_2H_4 (1:100), C_3H_6/C_3H_8 (1:1) and C_2H_4/C_2H_6 (1:1) at 298 and 318 K under a total pressure of 1 bar (Fig. 2c and Fig. S19).

$$S = \frac{x1/y1}{x2/y2}$$

Additional Gas Adsorption Isotherms for MFM-520

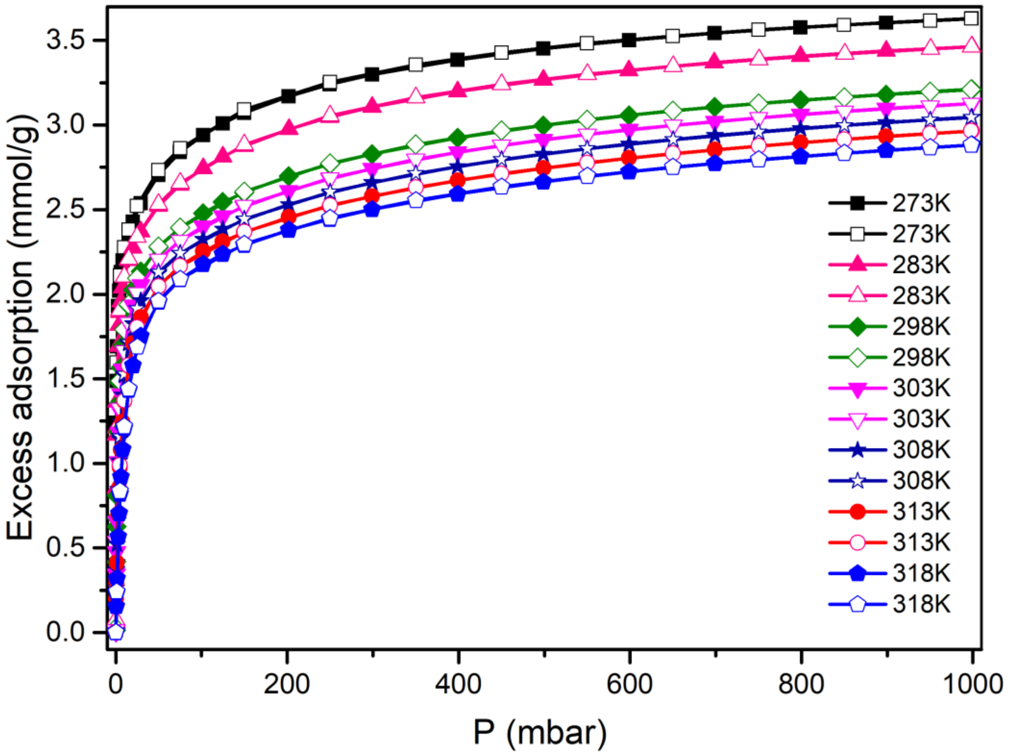

Fig. S1. Adsorption isotherms at different temperatures for C_2H_2 in MFM-520 (adsorption: solid; desorption: open).

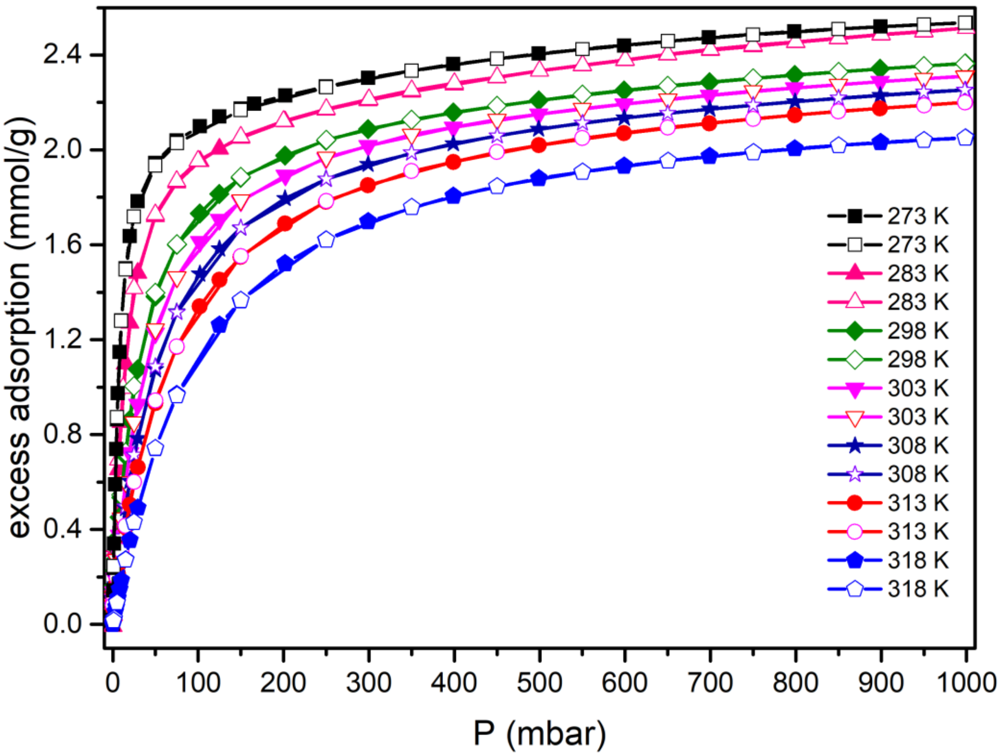

Fig. S2. Adsorption isotherms at different temperatures for C_2H_4 in MFM-520 (adsorption: solid; desorption: open).

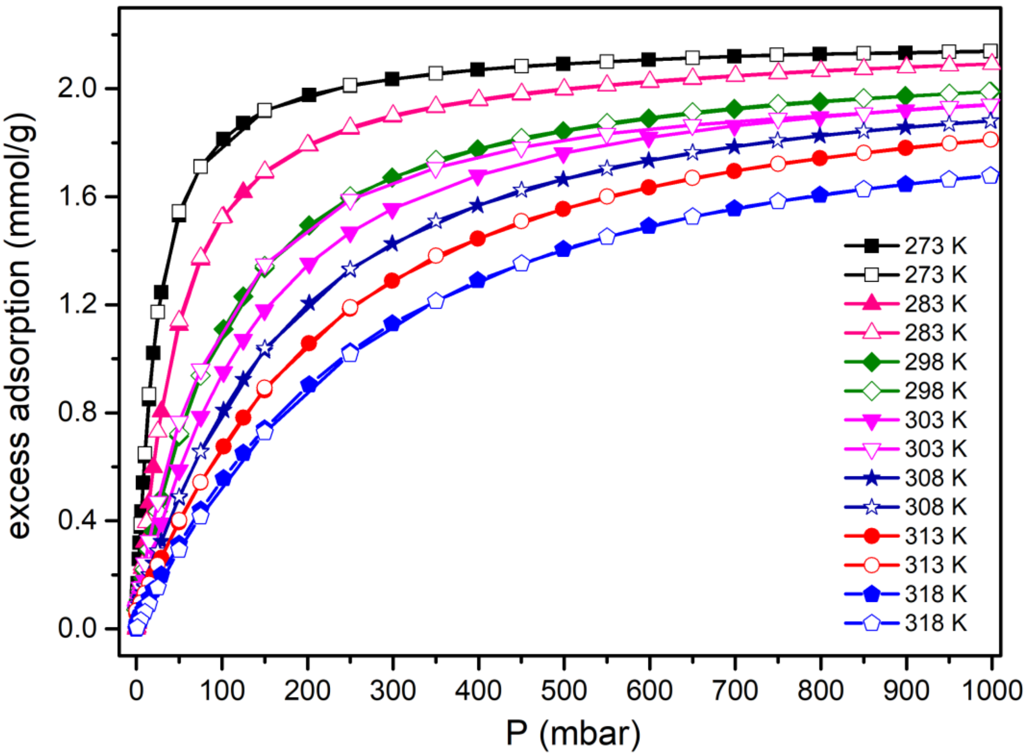

Fig. S3. Adsorption isotherms at different temperatures for C_2H_6 in MFM-520 (adsorption: solid; desorption: open).

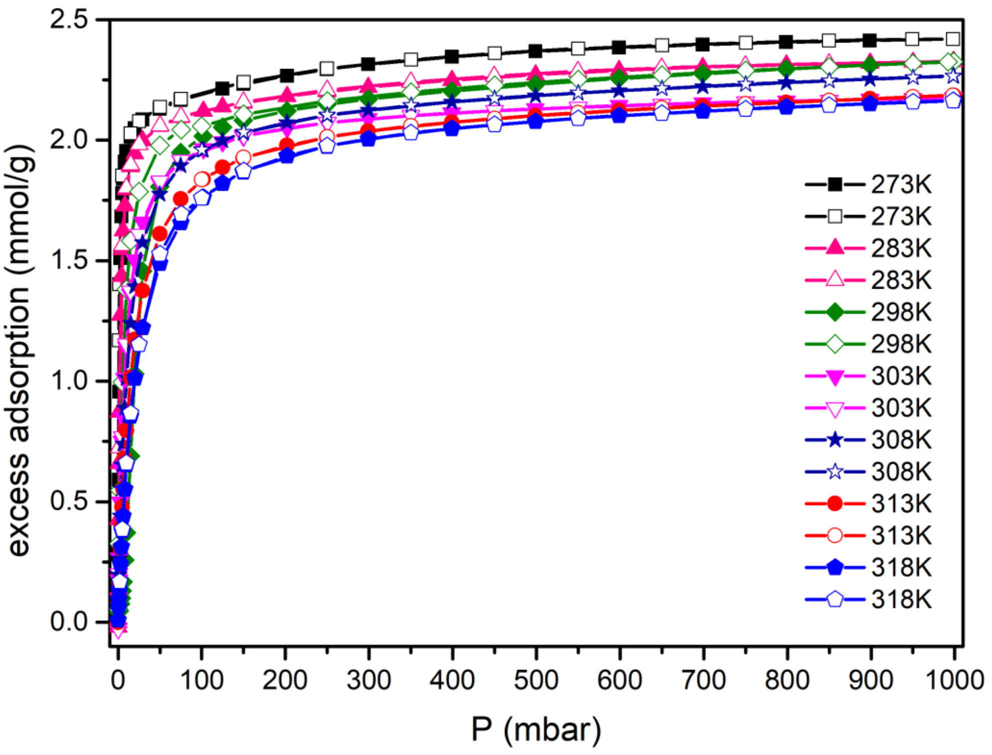

Fig. S4. Adsorption isotherms at different temperatures for C_3H_6 in MFM-520 (adsorption: solid; desorption: open).

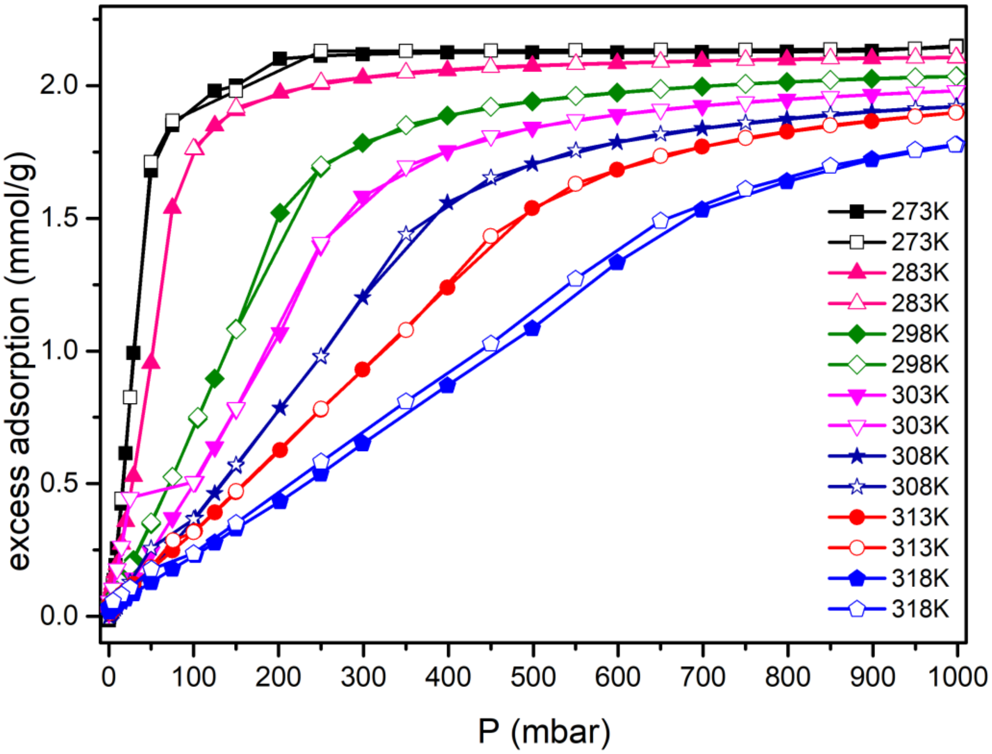

Fig. S5. Adsorption isotherms at different temperatures for C_3H_8 in MFM-520 (adsorption: solid; desorption: open).

Summary of Materials Reported for Olefin Purification

Table S1. Summary of selective MOFs for purification of olefins

Gas separation	MOF	BET (m^2/g)	Adsorption amount at 1 bar (mmol/g)	IAST Selectivity	Temperature (K)	Ref
C_3H_6/C_3H_8	MFM-520	330	2.33/2.03	23-16	318	**This work**
	Fe_2(dobdc)	1350	6.8/6.0	14	318	[11]
	KAUST-7	280	~1.4/<0.1	-	298	[12]
	Y-abtc	427[e]	1.9/<0.1	-	298	[13]
	Co(AIP)(BPY)$_{0.5}$	-	2.0/0.5	21	298	[14]
	[Zn_2(5-aip)$_2$(bpy)]·(DMF)·(H_2O)$_2$	-	2.0/0.5	18	298	[15]
	NJU-Bai8	1098	2.7/2.7	>4	298	[16]
	ELM-12	-	1.4/1.3	1.5	298	[17]
C_2H_4/C_2H_6	MFM-520	330	2.36/1.93	3.0	318	**This work**
	Fe_2(dobdc)	1350	6.2/5.1	13.6	318	[11]
	Fe_2(*m*-dobdc)	1295	6.9/5.9	＞25	298	[18]
	NOTT-300	1370	4.3/0.9	48.7	293	[19]
	USTA-280	331	2.5/<0.1	＞10^4 [g]	298	[20]
	SBA-15[a]	7.0	0.89/0.56	1.6	303	[21]
	Co-gallate	475	3.4/0.3	52	298	[22]
C_2H_2/C_2H_4	MFM-520	330	3.09/2.36	12[d]	318	**This work**
	MIL-100(Fe)	2300	5.3/3.0[c]	7-17	313	[23]
	UTSA-200a	612	3.7/0.63	6,000[d]	298	[24]
	ELM-11	-	3.7/<0.1	>10^4 [h]	298	[25]
	ELM-13	-	3.2/<0.1	>10^4 [h]	298	[25]
	SIFSIX-2-Cu-i	503	4.0/2.2	39-45[d]	298	[26]
	UTSA-67a	1137	5.1/2.8	5-6[d]	293	[27]
	Mn(II)-Mn(III)[f]	362[e]	3.2/0.24	16[d]	293	[28]
	ELM-12	-	2.6/1.0	14.8 [d]	298	[29]
	UTSA-300a	311	3.4/<0.1	>10^4 [d,g]	273	[30]
	USTA-100	970	4.3/1.7	10[d]	296	[31]

[a] no experimental breakthrough data; [b] uptake at 600 mbar; [c] uptake at 293K; [d] C_2H_2/C_2H_4 ratio 1:99; [e] BET measured by CO_2; [f] {[Mn_3(bipy)$_3$(H_2O)$_4$][Mn(CN)$_6$]$_2$·2(bipy)$_4H_2O$}$_n$; [h] These high selectivities are for qualitative comparisons only because the presence of molecular sieving effect has invalidated the use of IAST model.

Summary of Crystallographic Data for Hydrocarbon-loaded MFM-520

Table S2. Summary of crystallographic data

	MFM-520·C_2H_2	MFM-520·C_2H_4	MFM-520·C_2H_6	MFM-520·C_3H_6	MFM-520·C_3H_8
CCDC No.	1997960	1997961	1997963	1997962	1997964
Empirical formula	$C_{16.34}H_{6.34}N_2O_8Zn_2$	$C_{15.76}H_{7.52}N_2O_8Zn_2$	$C_{15.93}H_{9.79}N_2O_8Zn_2$	$C_{16.77}H_{9.55}N_2O_8Zn_2$	$C_{17}H_{12}N_2O_8Zn_2$
Crystal system	Tetragonal				
Crystal colour	colourless				
Temperature, (K)	273				
Space group, (Z)	$P4_2/mnm$ (2)				
Wavelength (Å)	0.7749	0.7749	0.7288	0.7749	0.7288
a<br>b<br>c (Å)	7.0469(15)<br>7.0469(15)<br>19.873(6)	7.0536(16)<br>7.0536(16)<br>19.790(6)	7.079(4)<br>7.079(4)<br>19.784(15)	7.0556(13)<br>7.0556(13)<br>19.847(5)	6.967(7)<br>6.967(7)<br>19.85 (3)
Density (g/cm^3)	1.647	1.631	1.634	1.673	1.734
Formula weight	489.44	486.63	487.97	497.84	503.03
Crystal size (mm^3)	0.100 x 0.020 x 0.005	0.100 x 0.020 x 0.005	0.100 x 0.100 x 0.020	0.100 x 0.020 x 0.005	0.100 x 0.100 x 0.020
2θ range (°)	3.344 to 22.792	3.343 to 24.357	3.134 to 18.452	3.341 to 22.793	3.178 to 21.373
Reflections collected	4607	5513	2436	4574	3176
R_1 [$I>2\sigma(I)$]	0.0884	0.0619	0.1897	0.0984	0.1843
$_wR_2$ (all data)	0.2710	0.1535	0.5938	0.2745	0.4845
Data / restraints / parameters	302/6/35	370/1/45	204/26/33	300/41/45	296/10/44
Goodness-of-fit on F^2	1.106	1.135	2.685	1.175	1.629
Independent reflections (R_{int})	302 (0.0597)	307 (0.0619)	204 (0.1093)	300 (0.0440)	296 (0.1854)

Isosteric Heats of Adsorption

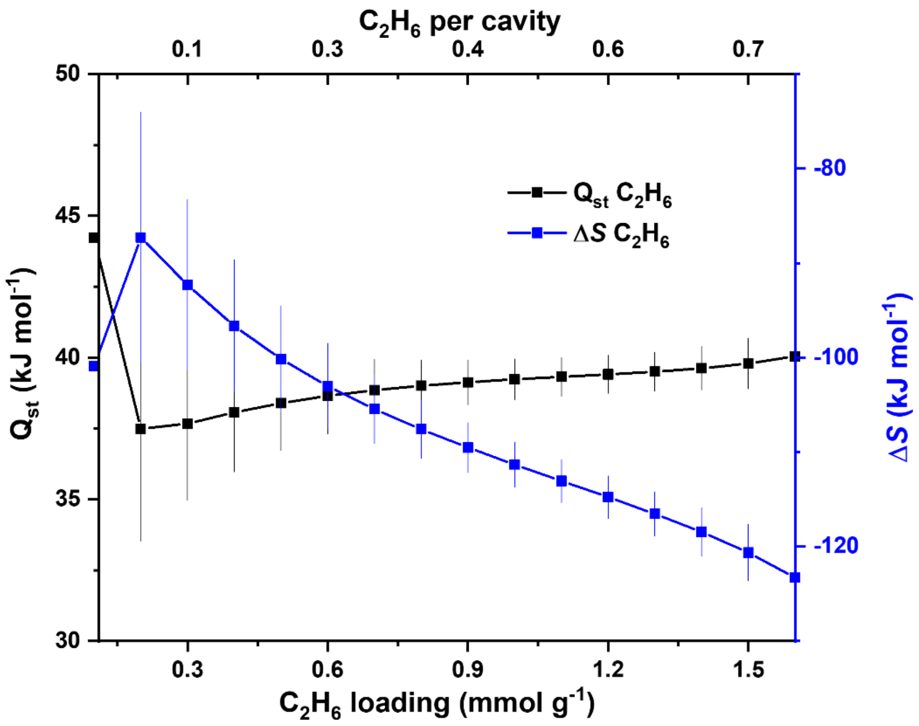

Fig. S6. Variation of isosteric heat of adsorption (Q_{st}) and entropy (ΔS) for C_2H_6 uptake in MFM-520.

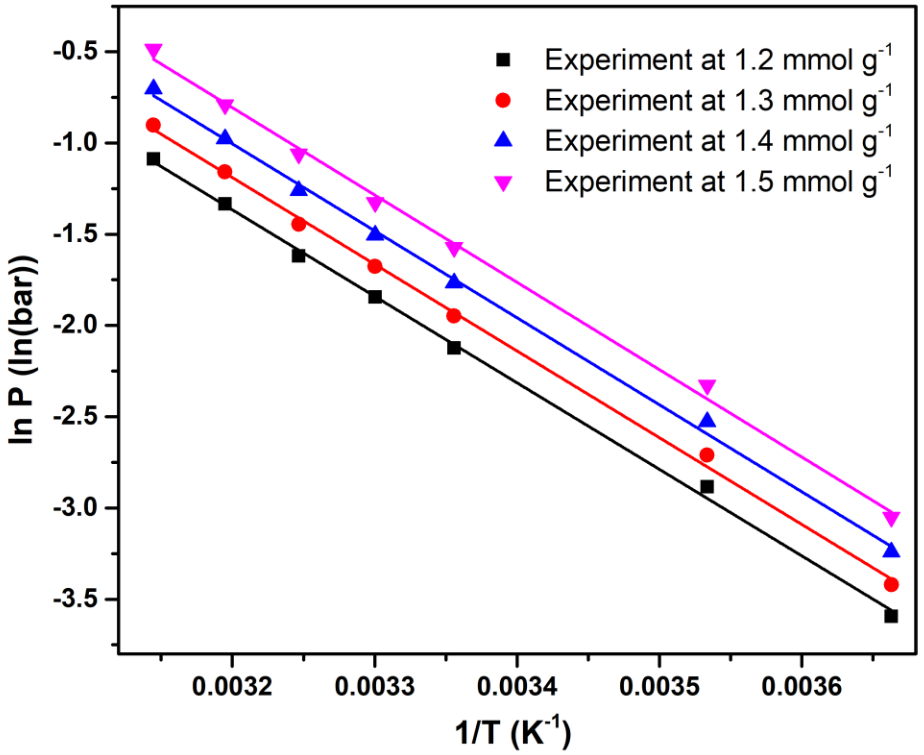

Fig. S7. Linear fitting of van't Hoff plots for the adsorption isotherms of C_2H_6 in MFM-520 at 1.2-1.5 mmol/g loading

Table S3. Thermodynamic parameters for adsorption of C_2H_6 in MFM-520

n (mmol g^{-1})	Q_{st} (kJ mol^{-1})	Q_{st} error	ΔS (J K^{-1} mol^{-1})	ΔS error	R^2
0.3	37.7	2.7	-92.3	9.1	0.9698
0.4	38.1	2.1	-96.7	7.0	0.9821
0.5	38.4	1.7	-100.1	5.6	0.9887
0.6	38.7	1.3	-103.0	4.5	0.9928
0.7	38.9	1.1	-105.4	3.7	0.9953
0.8	39.0	0.9	-107.6	3.0	0.9968
0.9	39.1	0.8	-109.5	2.6	0.9976
1.0	39.2	0.7	-111.3	2.4	0.9980
1.1	39.3	0.7	-113.0	2.3	0.9982
1.2	39.4	0.7	-114.8	2.3	0.9983
1.3	39.5	0.7	-116.5	2.3	0.9982
1.4	39.6	0.8	-118.4	2.5	0.9978
1.5	39.8	0.9	-120.6	3.0	0.9969
1.6	40.1	1.1	-123.3	3.8	0.9952

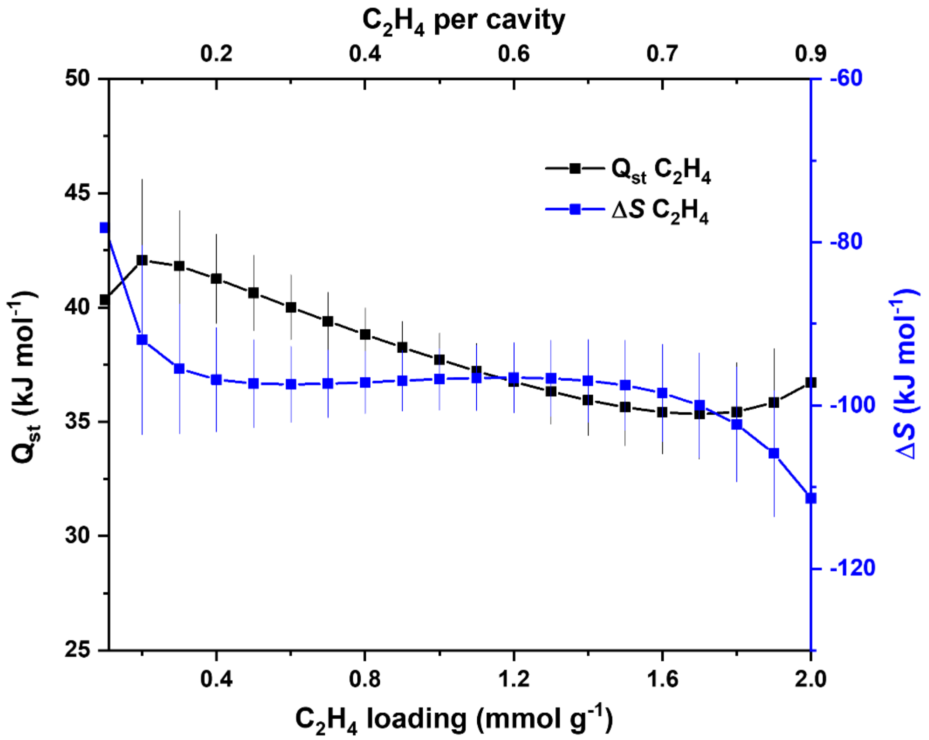

Fig. S8. Variation of isosteric heat of adsorption (Q_{st}) and entropy (ΔS) for uptake of C_2H_4 in MFM-520.

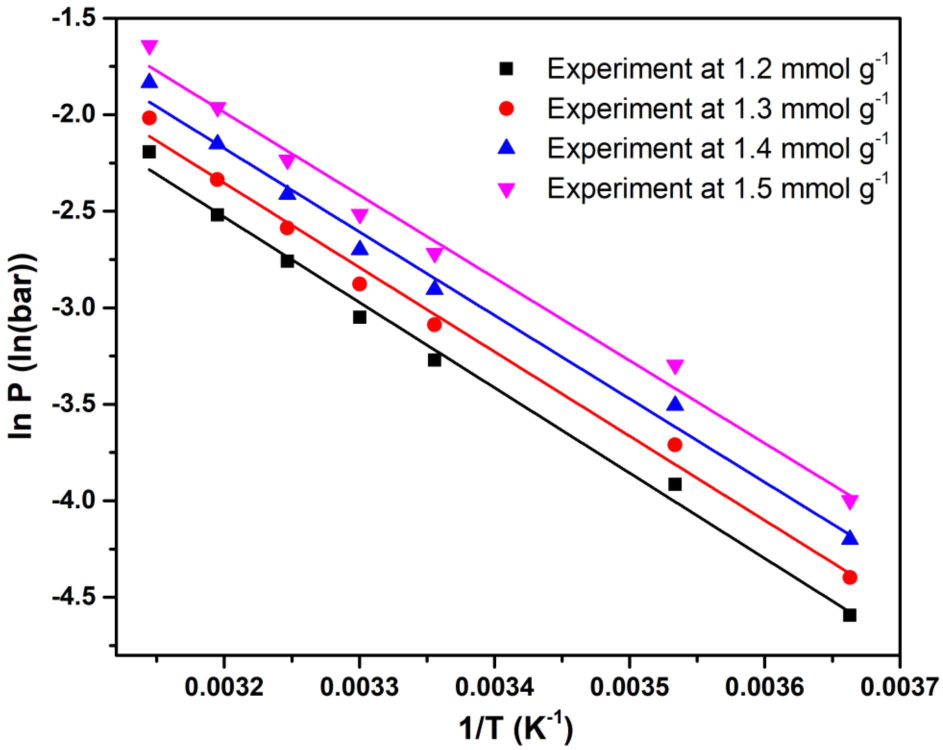

Fig. S9. Linear fitting of van't Hoff plots for adsorption isotherms of C_2H_4 in MFM-520 at 1.2-1.5 mmol/g loading.

Table S4. Thermodynamic parameters for adsorption of C_2H_4 in MFM-520.

n (mmol g^{-1})	Q_{st} (kJ mol^{-1})	Q_{st} error	ΔS (J K^{-1} mol^{-1})	ΔS error	R^2
0.3	41.8	2.4	-95.5	7.9	0.9803
0.4	41.3	1.9	-96.8	6.4	0.9869
0.5	40.6	1.6	-97.3	5.4	0.9904
0.6	40.0	1.4	-97.4	4.6	0.9926
0.7	39.4	1.3	-97.3	4.1	0.9939
0.8	38.8	1.2	-97.2	3.8	0.9946
0.9	38.3	1.1	-97.0	3.7	0.9948
1.0	37.7	1.1	-96.8	3.8	0.9945
1.1	37.2	1.2	-96.6	4.0	0.9937
1.2	36.8	1.3	-96.6	4.3	0.9925
1.3	36.3	1.4	-96.7	4.6	0.9910
1.4	35.9	1.5	-97.0	5.1	0.9891
1.5	35.6	1.7	-97.5	5.5	0.9869
1.6	35.4	1.8	-98.5	6.0	0.9845
1.7	35.3	2.0	-100.0	6.4	0.9818
1.8	35.4	2.1	-102.3	7.0	0.9787
1.9	35.8	2.3	-105.9	7.7	0.9748
2.0	36.7	2.7	-111.4	9.0	0.9678

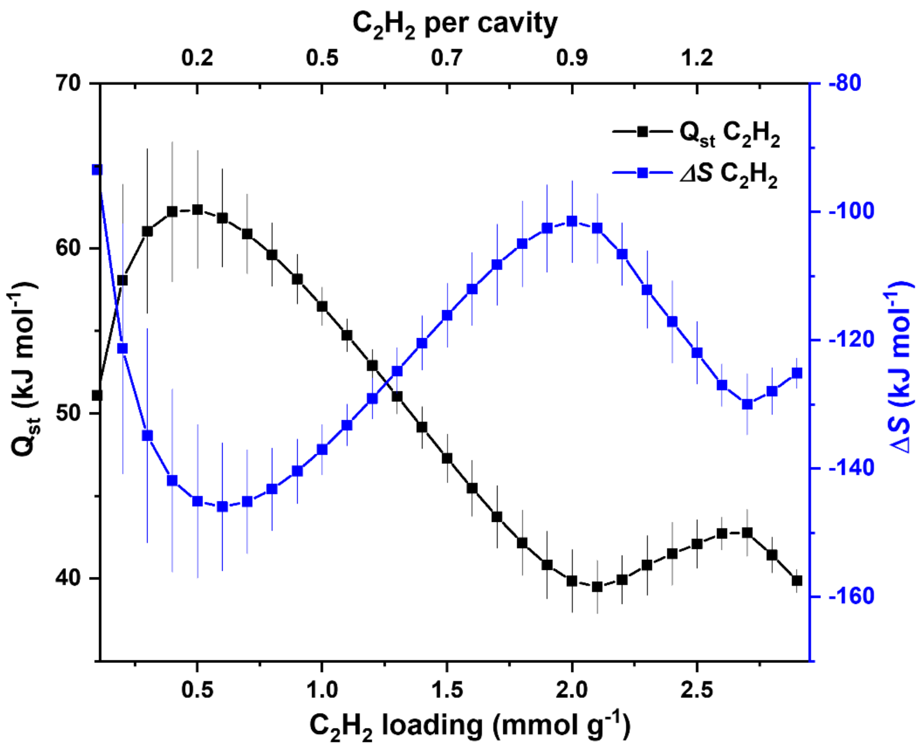

Fig. S10. Variation of isosteric heat of adsorption (*Q_{st}*) and entropy (*ΔS*) for uptake of C_2H_2 in MFM-520.

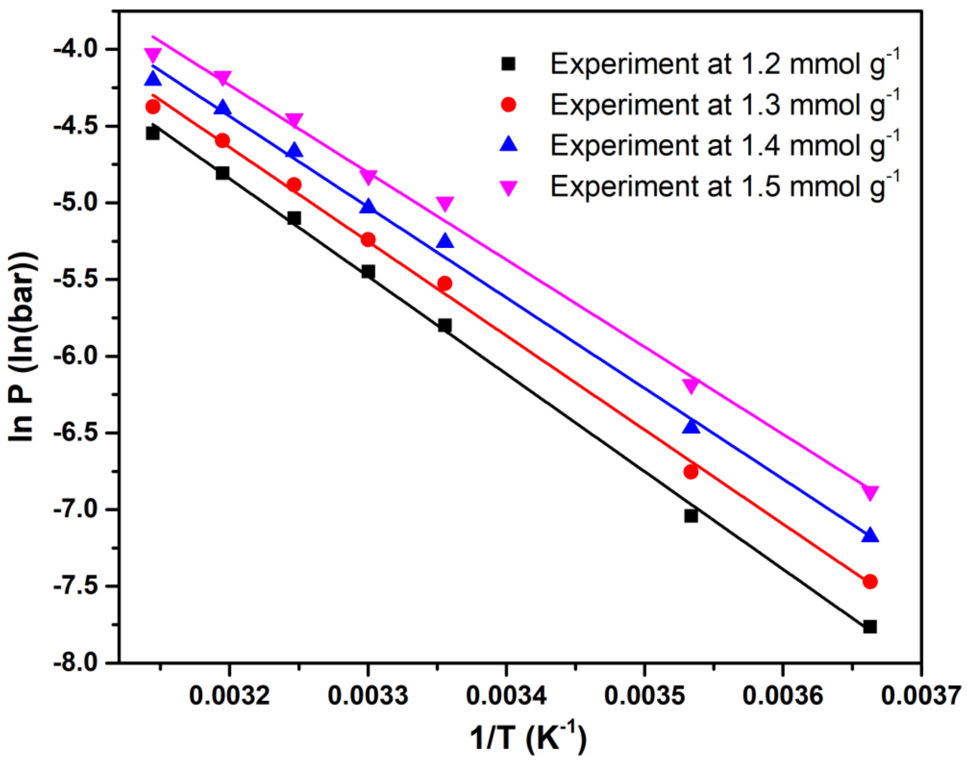

Fig. S11. Linear fitting of van't Hoff plots for adsorption isotherms of C_2H_2 in MFM-520 at 1.2-1.5 mmol/g loading.

Table S5. Thermodynamic parameters for adsorption of C_2H_2 in MFM-520.

n (mmol g^{-1})	Q_{st} (kJ mol^{-1})	Q_{st} error	ΔS (J K^{-1} mol^{-1})	ΔS error	R^2
0.3	61.1	5.0	-134.8	16.7	0.9614
0.4	62.2	4.2	-141.9	14.2	0.9727
0.5	62.4	3.6	-145.1	12.0	0.9806
0.6	61.8	3.0	-146.0	9.9	0.9864
0.7	60.9	2.4	-145.2	8.1	0.9907
0.8	59.6	1.9	-143.2	6.4	0.9939
0.9	58.1	1.5	-140.4	5.0	0.9961
1.0	56.5	1.2	-137.0	3.9	0.9975
1.1	54.7	1.0	-133.2	3.2	0.9982
1.2	52.9	0.9	-129.1	3.1	0.9981
1.3	51.0	1.1	-124.8	3.6	0.9974
1.4	49.2	1.3	-120.4	4.2	0.9961
1.5	47.3	1.5	-116.1	5.0	0.9941
1.6	45.5	1.7	-112.0	5.7	0.9917
1.7	43.7	1.9	-108.2	6.3	0.9891
1.8	42.2	2.0	-105.0	6.7	0.9868
1.9	40.8	2.0	-102.6	6.8	0.9855
2.0	39.9	1.9	-101.5	6.4	0.9865
2.1	39.5	1.6	-102.6	5.4	0.9900
2.2	39.9	1.5	-106.6	4.9	0.9920
2.3	40.8	1.8	-112.1	6.0	0.9884
2.4	41.5	1.9	-117.1	6.4	0.9875
2.5	42.1	1.5	-122.0	4.9	0.9929
2.6	42.7	1.0	-127.0	3.3	0.9968
2.7	42.8	1.4	-130.0	4.7	0.9935
2.8	41.4	1.1	-128.0	3.6	0.9960
2.9	39.9	0.7	-125.1	2.4	0.9981

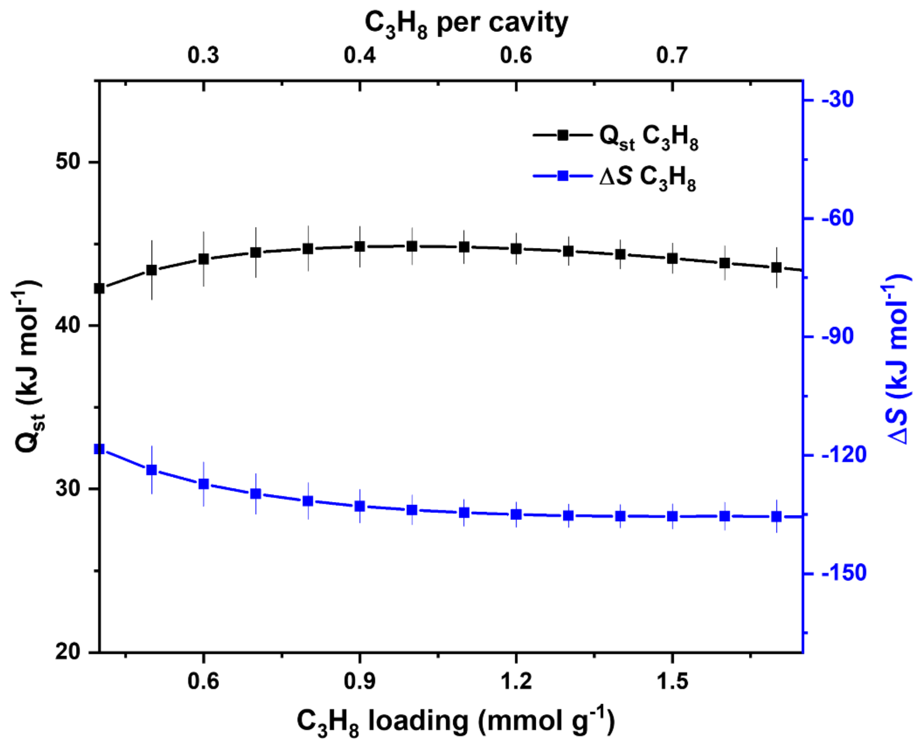

Fig. S12. Variation of isosteric heat of adsorption (Q_{st}) and entropy (ΔS) for uptake of C_3H_8 in MFM-520.

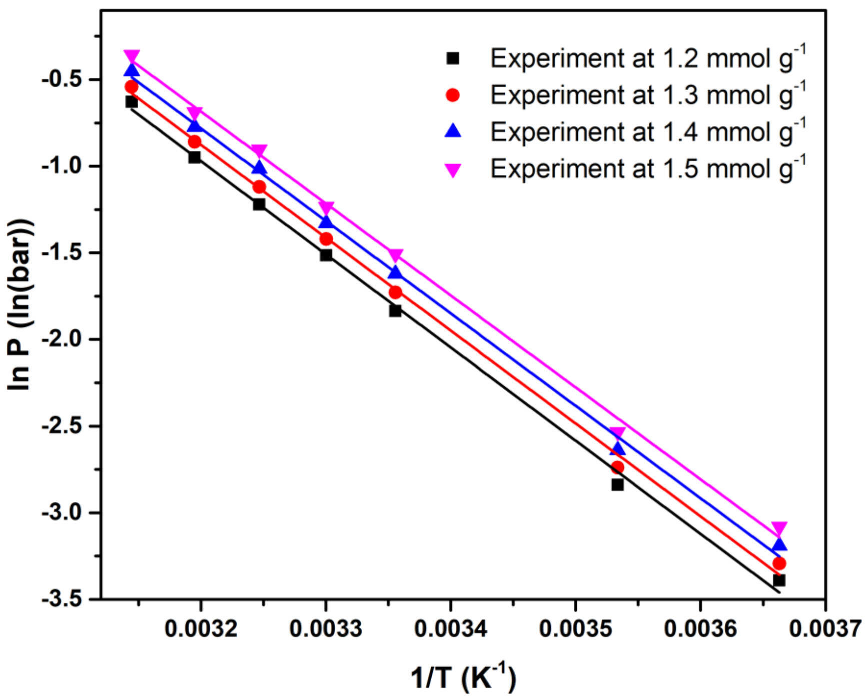

Fig. S13. Linear fitting of Van't Hoff plots for adsorption isotherms of C_3H_8 in MFM-520 at 1.2-1.5 mmol/g loading.

Table S6. Thermodynamic parameters for adsorption of C_3H_8 adsorption in MFM-520.

n (mmol g^{-1})	*Qst* (kJ mol^{-1})	*Qst* error	*ΔS* (J K^{-1} mol^{-1})	*ΔS* error	R^2
0.4	42.3	2.0	-118.4	6.6	0.9872
0.5	43.4	1.8	-123.7	6.1	0.9897
0.6	44.1	1.7	-127.3	5.6	0.9915
0.7	44.5	1.5	-129.8	5.1	0.9930
0.8	44.7	1.4	-131.6	4.6	0.9943
0.9	44.8	1.2	-132.9	4.2	0.9954
1.0	44.9	1.1	-133.9	3.8	0.9962
1.1	44.8	1.0	-134.6	3.4	0.9969
1.2	44.7	0.9	-135.0	3.1	0.9974
1.3	44.6	0.9	-135.3	3.0	0.9976
1.4	44.4	0.9	-135.5	2.9	0.9977
1.5	44.1	0.9	-135.5	3.1	0.9974
1.6	43.8	1.0	-135.5	3.5	0.9966
1.7	43.6	1.2	-135.5	4.1	0.9952
1.8	43.2	1.7	-135.7	5.9	0.9920

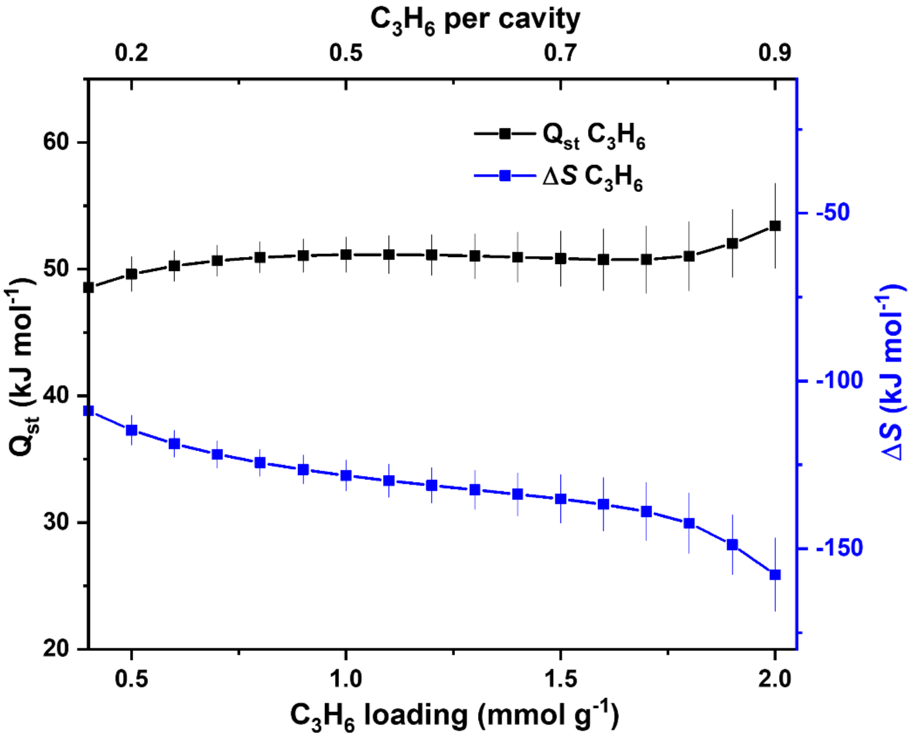

Fig. S14. Variation of isosteric heat of adsorption (Q_{st}) and entropy (ΔS) for uptake of C_3H_6 in MFM-520.

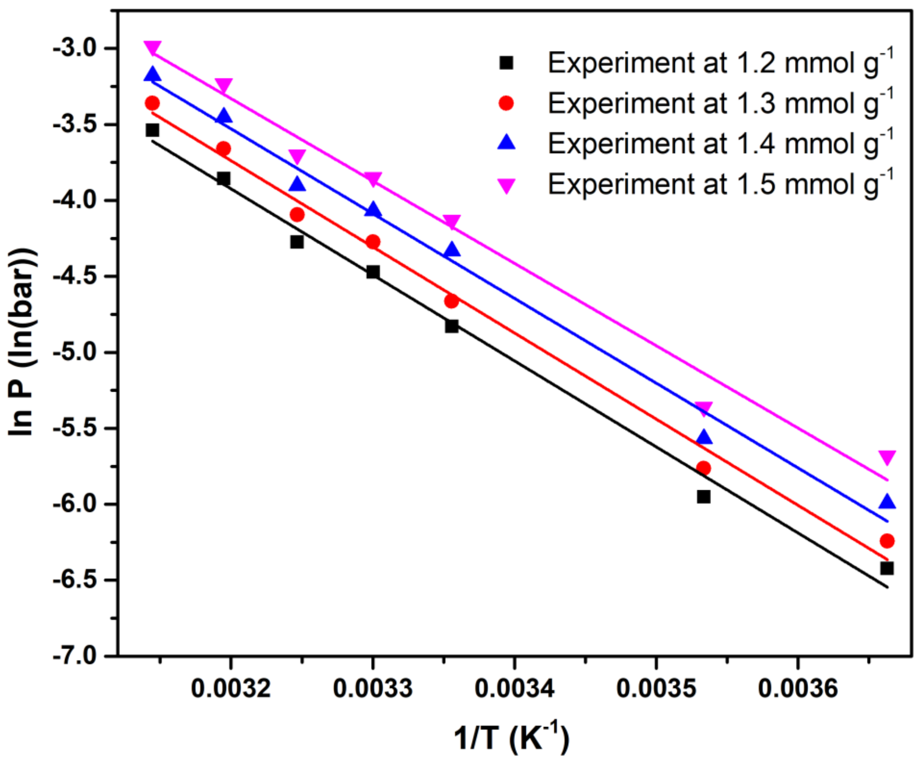

Fig. S15. Linear fitting of van't Hoff plots for the adsorption isotherms of C_3H_6 in MFM-520 at 1.2-1.5 mmol/g loading.

Table S7. Thermodynamic parameters for adsorption of C_3H_6 in MFM-520.

n (mmol g^{-1})	*Qst* (kJ mol^{-1})	*Qst* error	*ΔS* (J K^{-1} mol^{-1})	*ΔS* error	R^2
0.4	48.5	1.8	-108.9	-5.8	0.9947
0.5	49.6	1.3	-114.6	-4.4	0.9971
0.6	50.3	1.2	-118.7	-4.0	0.9977
0.7	50.7	1.2	-121.8	-3.9	0.9978
0.8	50.9	1.2	-124.3	-4.1	0.9976
0.9	51.1	1.3	-126.4	-4.3	0.9974
1.0	51.1	1.4	-128.2	-4.6	0.9971
1.1	51.2	1.5	-129.7	-4.9	0.9966
1.2	51.1	1.6	-131.1	-5.3	0.9961
1.3	51.0	1.8	-132.5	-5.8	0.9953
1.4	50.9	2.0	-133.8	-6.4	0.9941
1.5	50.8	2.2	-135.1	-7.2	0.9927
1.6	50.8	2.4	-136.8	-8.0	0.9909
1.7	50.8	2.6	-138.9	-8.7	0.9892
1.8	51.0	2.7	-142.4	-9.0	0.9886
1.9	52.0	2.7	-148.8	-8.8	0.9895
2.0	53.4	3.3	-157.7	-10.9	0.9846

PXRD Analysis of PIM-1/Matrimid/MFM-520 (w/w/w =10: 10: 1) MMM

PXRD analysis of the membrane was conducted on a Philips X'Pert XRD using Cu-Kα radiation to confirm the retention of the crystal structure of the MOF after incorporation into the MMM.

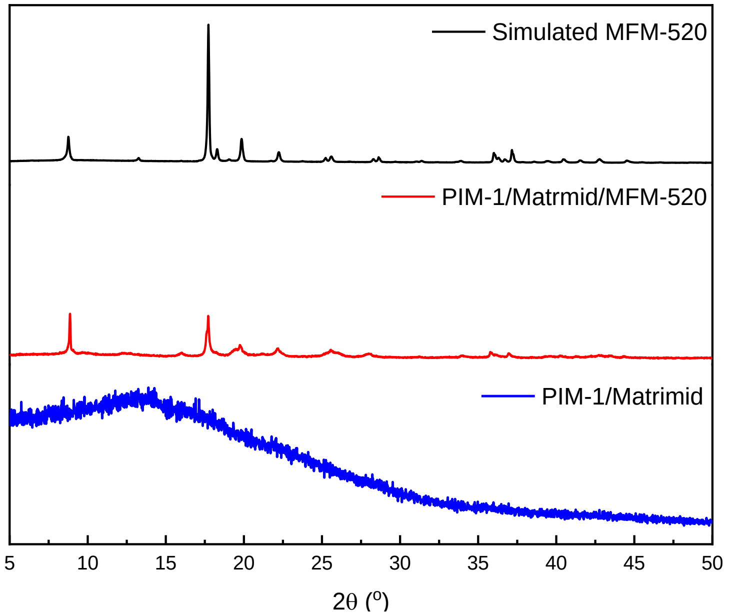

Fig. S16. Comparison of PXRD patterns of the bare polymer, MFM-520 and of the MMM.

SEM Analysis of PIM-1/Matrimid/MFM-520 (*w/w/w* = 10:10:1) MMM

Analysis of cross-sectioned membrane was conducted using scanning electron microscopy (SEM). The MMM was cross-sectioned mechanically and adhered to the sample stub *via* a carbon tab. The deformed region highlighted by the orange square in Fig. S17a contains surface dips from mechanical damage induced during cross-sectioning. The undeformed region, as highlighted *via* the blue square in Fig. S17a, is homogeneous in texture. Fig. S17b shows a magnified image of this undeformed area. Even in the deformed MMM region seen in Fig. S17c, unperturbed areas, such as the green square highlighted and magnified *via* Fig. S17d, show further homogeneous textures within the MMM. The images were acquired at 15 kV with spotsize 3.5 on the Quanta 650 FEG SEM at magnifications ranging from 1000X to 26,000X.

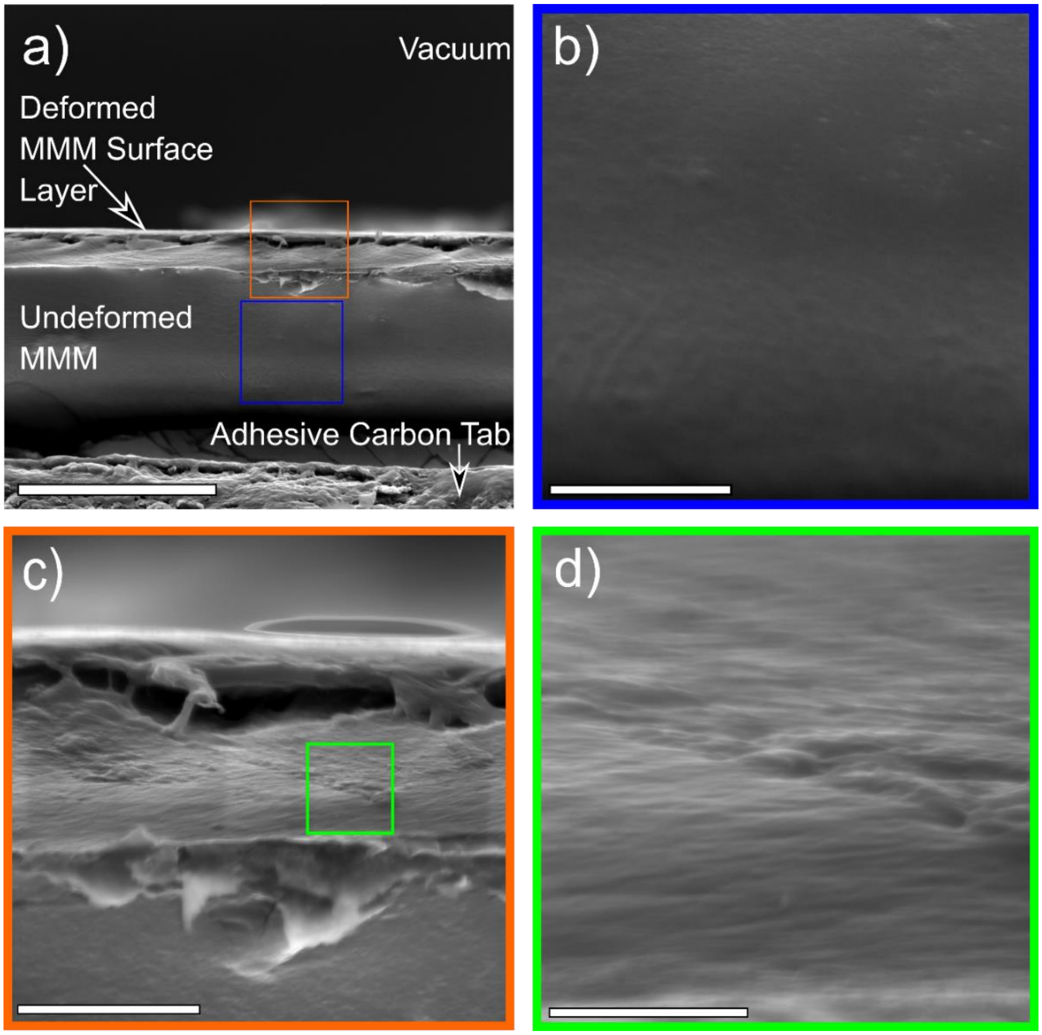

Fig. S17. Cross-sectional SEM images of the PIM-1/Matrimid/MFM-520 MMM. (**a**) Low-magnification image showing both the deformed region (orange square) from mechanical damage and undeformed region (blue square). (**b**) Magnified image of the undeformed area highlighted *via* the blue square in (**a**). (**c**) Magnified image of the deformed area highlighted via the orange square in (**a**), showing mechanical damage. (**d**) Further magnified image of the area highlighted via the green square in (**c**), highlighting further homogeneous texture. Scale bars are 50, 10 and 2 µm for (**a**), (**b**) and (**c**), and (**d**), respectively.

Optical Photographs of Fabricated Membranes

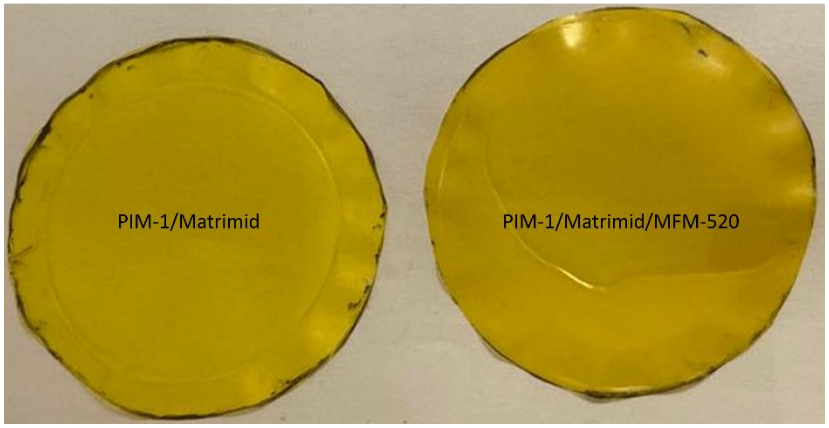

Fig. S18. Optical photographs of the MMM based upon PIM-1/Matrimid and PIM-1/Matrimid/MFM-520.

The Reproducibility of the Separation Performance of the PIM-1/Matrimid/MFM-520 MMM

Table S8. Performance of C_3H_6/C_3H_8 separation of the PIM-1/Matrimid/MFM-520 MMM.

PIM-1/Matrimid/MFM-520 MMM batch	Cycle	C_3H_6 Permeability (Barrer)	C_3H_6/C_3H_8 Separation Factor
**1**	1	1980	8.5
	2	2187	7.2
	3	2084	7.8
**2**	1	2010	7.8
	2	1990	7.9
	3	1897	8.9
**3**	1	1898	7.7
	2	1916	7.5
	3	1890	7.5
**Average**	-	1984.5(6)	7.8(7)

The Reproducibility of the Separation Performance of the PIM-1/Matrimid Membrane

Table S9. Performance of C3H6/C3H8 separation of the PIM-1/Matrimid Membrane

PIM-1/Matrimid Membrane batch	Cycle	C_3H_6 Permeability (Barrer)	C_3H_6/C_3H_8 Separation Factor
**1**	1	3250	4.7
	2	3200	4.8
	3	3105	4.1
**2**	1	3283	4.2
	2	3254	4.6
	3	3400	5.3
**3**	1	3130	4.4
	2	3250	4.3
	3	3300	3.9
**Average**	-	3241.9(2)	4.4(7)

Summary of Selected C_3H_6/C_3H_8 Separations using Membranes

Table S10. C_3H_6/C_3H_8 separation performance of selective reported membranes

Membrane	Temperature (K)	Feed Pressure (bar)	$P_{(C3H6)}$ (Barrer)	C_3H_6/C_3H_8 selectivity	Ref
PIM-1/Matrimid	298	1.5	3241	4.4	This work
PIM-1/Matrimid/MFM-520	298	1.5	1984	7.8	This work
Matrimid®	298	2-3	0.1	16	[32]
Matrimid®-Thermid 85/15	298	2-3	0.03	4	[32]
Pyralin 2566	298	2-3	0.09	21	[32]
PPO	303	2-4	9	4.25	[33]
EC	303	3-3.9	52[a]	3.25[a]	[34]
CA	303	3-3.9	15.2[a]	2.6[a]	[34]
PSF	303	3-3.9	25[a]	1.4[a]	[34]
6FDA-mPD	308	3.8	0.13	10	[35]
6FDA-IpDA	308	3.8	0.58	15	[35]
6FDA-6FpDA	308	3.8	0.89	16	[35]
Matrimid®	308	2	0.1	10	[36]
6FDA-33'DMDB	308	1.1	0.15	13.2	[36]
6FDA-TeMPD	323	2	37	8.6	[37]
BPDA-TeMPD	323	2	3.2	13	[37]
PPO	323	2	2.3	9.1	[37]
P4MP	323	2	54	2	[37]
1.2PB	323	2	260	1.7	[37]
PDMS	323	2	6600	1.2	[37]
6FDA-ODA	373	2	0.48	11	[37]
6FDA-TrMPD	323	2	30	11	[37]
6FDA-DDBT	323	2	1.8	20	[38]
6FDA-DDBT	323	2	0.76	27	[37]
SIFSIX-3-Zn/PIM-1	308	1	4012[b]	7.9[b]	[10]
ZIF-8/XLPEO	308	2	28.5	15	[39]
ZIF-8/6FDA-Durene	308	2	37.7[b]	20.7[b]	[40]
ZIF-67/6FDA-Durene	308	2	34.1[b]	29.9[b]	[40]
ZIF-8/6FDA-DAM	308	2	56.2[b]	31[b]	[41]
ZIF-8/6FDA-Durene/DABA	308	3.5	47.3[b]	27.4[b]	[42]
AgPF4/Pebax	303	2-6	123.3	20.4	[43]
CC3/6FDA-DAM	293	3	480[b]	15.4[b]	[44]
CC3/6FDA-DAM	293	3	390[b]	12.1[b]	[44]
Carbon PAEK/Azide (50:50) - 450℃	308	3.5	17[b]	31[b]	[45]
Carbon PAEK/Azide (80:20) - 450℃	308	3.5	46[b]	17[b]	[45]
Carbon PAEK/Azide (50:50) - 550℃	308	3.5	16[b]	48[b]	[45]
Carbon PAEK/Azide (80:20) - 550℃	308	3.5	48[b]	44[b]	[45]
Carbon PAEK/Azide (50:50) - 650℃	308	3.5	6.6[b]	24[b]	[45]
Carbon PAEK/Azide (80:20) - 650℃	308	3.5	4.4[b]	16[b]	[45]

[a] Mixed gas results: 55% propylene/45% propane feed; [b] The values were obtained from single-gas permeation tests.

IAST analysis at 298 K

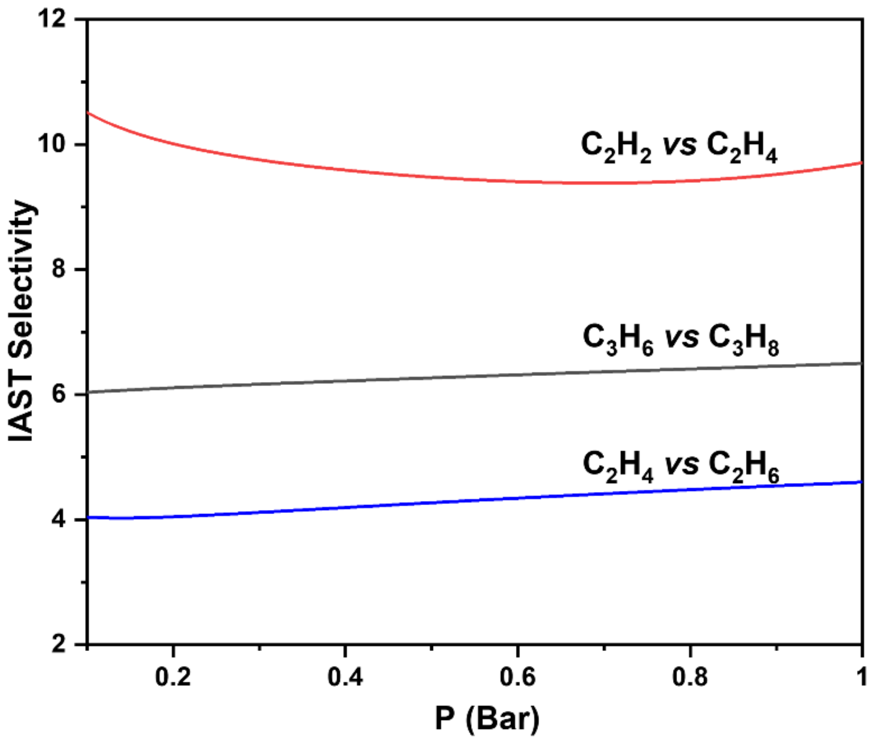

Fig S19. IAST selectivities for equimolar mixtures of C_2H_4/C_2H_6 and C_3H_6/C_3H_8, and of a 1:100 mixture of C_2H_2/C_2H_4 at 0.1-1.0 bar using MFM-520 at 298K.

View of packing of adsorbed C_2H_2 molecules in MFM-520

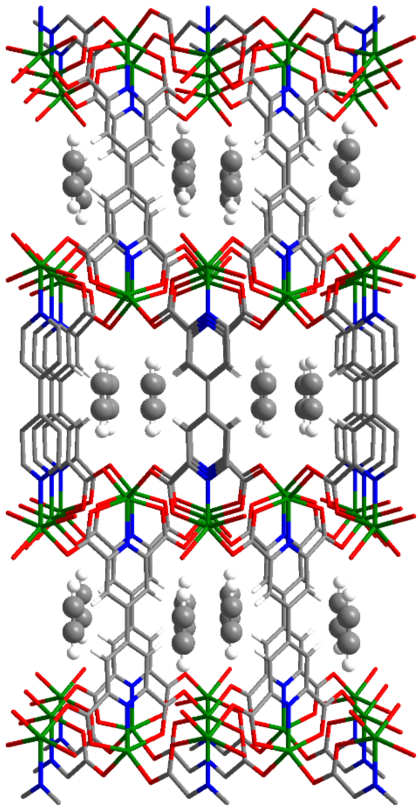

Fig S20. View along the a-axis of packing of trapped C_2H_2 molecules within MFM-520 (C: grey; N: blue; O: red; H: white; Zn: dark green).

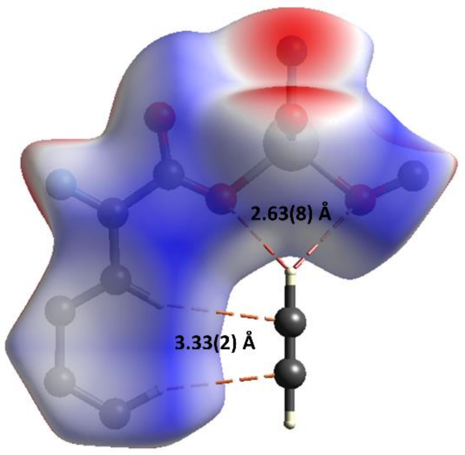

Fig S21. View of the region of interaction between C_2H_2 and MFM-520 by Hirshfeld surface analysis[46]

SI References

[1] X. Lin, A. J. Blake, C. Wilson, X. Sun, N. R. Champness, M. W. George, P. Hubberstey, R. Mokaya, M. Schröder, *J. Am. Chem. Soc.* **2006**, 128, 10745–10753.

[2] G. Kresse, J. Furthmüller, *Phys. Rev. B*. **1996**, 54, 11169–11186.

[3] P. E. Blöchl, *Phys. Rev. B*. **1994**, 50, 17953–17979.

[4] G. Kresse, D. Joubert, *Phys. Rev. B*. **1999**, 59, 1758–1775.

[5] J. P. Perdew, K. Burke, M. Ernzerhof, *Phys. Rev. Lett.* **1996**, 77, 3865–3868.

[6] J. Klimeš, D. R. Bowler, A. Michaelides, *J. Phys. Condens. Matter*. **2009**, 22, 22201.

[7] A. Togo, I. Tanaka, *Scr. Mater.* **2015**, 108, 1–5.

[8] Y. Q. Cheng, L. L. Daemen, A. I. Kolesnikov, A. J. Ramirez-Cuesta, *J. Chem. Theory Comput.* **2019**, 15, 1974–1982.

[9] P. M. Budd, B. S. Ghanem, S. Makhseed, N. B. McKeown, K. J. Msayib, G. E. Tattersgall, *Chem. Commun.* **2004**, 4, 230–231.

[10] Q. Shen, S. Cong, R. He, Z. Wang, Y. Jin, H. Li, X. Cao, J. Wang, B. Bruggen, Y. Zhang, *J. Memb. Sci.* **2019**, 588, 117201.

[11] E. D. Bloch, W. L. Queen, R. Krishna, J. M. Zadrozny, C. M. Brown, J. R. Long, *Science* **2012**, 335, 1606–1611.

[12] A. Cadiau, K. Adil, P. M. Bhatt, Y. Belmabkhout, M. Eddaoudi, *Science.* **2016***,* 353, 137-140 .

[13] H. Wang, X. Dong, V. Colombo, Q. Wang, Y. Liu, W. Liu, X. Wang, D. Proserpio, A. Sironi, Y. Han, J. Li, *Adv. Mater.* **2018**, 30, 1–9.

[14] H. Wu, Y. Yuan, Y. Chen, F. Xu, D. Lv, Y. Wu, Z. Li, Q. Xia, *AIChE J.* **2019**, 1–8.

[15] Y. Chen, H. Wu, D, Lv, N. Yuan, Q. Xia, Z. Li, *Sep. Purif. Technol.* **2018**, 204, 75–80.

[16] X. Wang, R. Krishna, L. Li, B. Wang, T. He, Y. Zhang, J. Li, J. Li, *Chem. Eng. J.* **2018**, 346, 489–496.

[17] L. Li, R. Lin, X. Wang, W. Zhou, L. Jia, J, Li, B. Chen, *Chem. Eng. J.* **2018**, 354, 977–982.

[18] J. E. Bachman, M. T. Kapelewski, D. A. Reed, M. I. Gonzalez, J. R. Long, *J. Am. Chem. Soc.* **2017**, 139, 15363–15370.

[19] S. Yang, A. J. Ramirez-Cuesta, R. Newby, V. Garcia-Sakai, P. Manuel, S. Callear, S. Campbell, C. Tang, M. Schröder, *Nat. Mater*. **2015**, 7, 121–129.

[20] R. Lin, L. Li, H. Zhou, H. Wu, C. He, S. Li, R. Krishna, J. Li, W. Zhou, B. Lin, *Nat. Mater.* **2018**, 17, 1128–1133.

[21] B. L. Newalkar, N. V Choudary, P. Kumar, S. Komarneni, T. S. G. Bhat, *Chem. Mater.* **2002**, 14, 304–309.

[22] Z. Bao, J. Wang, Z. Zhang, H. Xing, Q. Yang, Y. Yang, H. Wu, R. Krishna, W. Zhou, B. Chen, Q. Ren. *Angew. Chem. Int. Ed.* **2018**, 130, 16252–16257.

[23] H. S. Scott, M. Shivama, A. Bajpai, D. Madden, K. Chen, T. Pham, K. Forrest, A. Hogan, B. Space, J. Perry, M. J. Zaworotko, *ACS Appl. Mater. Interfaces*. **2017**, 9, 33395–33400.

[24] L. Li, H. Wen, C. He, R. Lin, R. Krishna, H. Wu, W. Zhou, J. Li, B. Li, B. Chen, *Angew. Chem. Int. Ed.* **2018**, 57, 15183–15188.

[25] L. Li, R. Krishna, Y. Wang, X. Wang, J. Yang, J. Li, *Eur. J. Inorg. Chem.* **2016**, 4457–4462.

[26] X. Cui, K. Chen, H. Xing, Q. Yang, R. Krishna, Z. Bao, W. Zhou, X. Dong, Y. Han, B. Li, Q. Ren, M. J. Zaworotko, B. Chen, *Science* **2016**, 353, 141–144.

[27] H. Wen, N. Li, H. Wang, R. Krishna, B. Chen, *Chem. Commun.* **2016**, 52, 1166–1169.

[28] A. Hazra, S. Jana, S. Bonakala, S. Balasubramanian, T. K. Maji, *Chem. Commun.* **2017**, 53, 4907–4910.

[29] L. Li, R. Lin, R. Krishna, X. Wang, B. Li, H. Wu, J. Li, W. Zhou, B. Chen, *J. Mater. Chem. A*. **2017**, 5, 18984–18988.

[30] R. Lin, L. Li, H. Wu, H. Arman, B. Li, R. Lin, W. Zhou, B. Chen, *J. Am. Chem. Soc.* **2017**, 139, 8022–8028.

[31] T. Hu, H. Wang, B. Li, R. Krishna H. Wu, W. Zhou, Y. Zhou, Y. Han, X. Wang, W. Zhu, Z. Yao, S. Xiang, B. Chen, *Nat. Commun.* **2015**, 6, 7328.

[32] J. J. Krol, M. Boerrigter, G. H. Koops, *J. Memb. Sci.* **2001**, 184, 275–286.

[33] S. Bai, S. Khan, *J. Memb. Sci.* **1998**, 147, 131–139.

[34] B. Yuan, H. Sun, T. Wang, Y. Xu, P. Li, Y. Kong, Q. Niu, *Sci. Rep.* **2016**, 6, 1–11.

[35] C. Staudt-Bickel, W. J. Koros, *J. Memb. Sci.* **2000**, 170, 205–214.

[36] R. L. Burns, W. J. Koros, *J. Memb. Sci.* **2003**, 211, 299–309.
[37] K. Tanaka*, J. Memb. Sci.* **1996**, 121, 197–207.
[38] K. Okamoto, K. Noborio, J. Hao, K. Tanaka, H. Kita, *J. Memb. Sci.* **1997**, 134, 171–179.
[39] D. Liu, L. Xiang, H. Chang, K. Chen, C. Wang, Y. Pan, Y. Li, Z. Jiang, *Chem. Eng. Sci.* **2019**, 204, 151–160.
[40] H. An, S. Park, H. Kwon, H. Jeong, J. Lee, *AIChE Annu. Meet.* **2017**, 3, 1362–1371.
[41] C. Zhang, Y. Dai, J. R. Johnson, O. Karvan, W. J. Koros. *J. Memb. Sci.* **2012**, 389, 34–42.
[42] M. Askari, T. S. Chung, *J. Memb. Sci.* **2013**, 444, 173–183.
[43] R. Murali, K. Rani, T. Sankarshana, A. F. Ismail, S. Sridhar, *Oil Gas Sci. Technol.* **2015**, 70, 381–390.
[44] Q. Zhang, H. Li, S. Chen, J. Duan, W. Jin, *J. Memb. Sci.* **2020**, 611, 118288.
[45] M. L. Chng, Y. Xiao, T. Chung, M. Toriida, S. Tamai, *Carbon* **2009**, 47, 1857–1866.
[46] M. J. Turner, J. J. McKinnon, S. K. Wolff, D. J. Grimwood, P. R. Spackman, D. Jayatilaka and M. A. Spackman, *CrystalExplorer17* ,**2017**. University of Western Australia.
